# Nutritional and health benefits of a partial substitution of red and processed meat with non-soy legumes: a 6-week randomized controlled trial in healthy working-age men

**DOI:** 10.1007/s00394-025-03783-x

**Published:** 2025-08-19

**Authors:** Sari Bäck, Essi Päivärinta, Tiina Pellinen, Suvi T. Itkonen, Mikko Lehtovirta, Maijaliisa Erkkola, Niina E. Kaartinen, Satu Männistö, Anne-Maria Pajari

**Affiliations:** 1 Department of Food and Nutrition, University of Helsinki, Helsinki, Finland; 2 Institute for Molecular Medicine Finland, University of Helsinki, Helsinki, Finland; 3 Department of Public Health, Finnish Institute for Health and Welfare, Helsinki, Finland

**Keywords:** Pulses, Iodine, Iron, Vitamin B12, Nutritional status, BeanMan study

## Abstract

**Purpose:**

Legumes are recommended as a sustainable alternative protein source to red and processed meat (RPM). We studied the effects of a partial substitution of RPM with non-soy legumes on nutrient intakes and status, anthropometric measurements, and biomarkers of cardiovascular diseases and type 2 diabetes risk in healthy working-age men.

**Methods:**

In a 6-week clinical trial, 102 men (mean age 38 years, range 21–61) were randomized to either the MEAT group, consuming 760 g/week of RPM (25% of total protein intake (TPI)) or the LEGUME group, consuming 200 g/week of RPM corresponding to the maximum of the Planetary Health Diet (5% of TPI) along with legume-based foods containing protein equivalent to 560 g/week of red meat (20% of TPI). TPI target was 18 E%. Four-day food records, blood, and 24-h urine samples were collected at baseline and endpoint.

**Results:**

At the endpoint, the LEGUME group had higher intakes of fiber (g/MJ, *P* = 0.006), polyunsaturated fatty acids (E%, *P* < 0.001), and iron (mg/MJ, *P* < 0.001) compared to the MEAT group, but lower intakes of saturated fatty acids (E%, *P* = 0.012) and vitamin B12 (µg/MJ, *P* < 0.001). The LEGUME group had lower vitamin B12 status (holotranscobalamin, *P* = 0.022), iodine status (24-h urinary iodine excretion, *P* = 0.041), total and LDL cholesterol (both *P* < 0.001), and BMI and weight (both *P* = 0.009).

**Conclusion:**

Implementing a moderate dietary shift by consuming more legumes and less RPM may beneficially affect biomarkers of cardiovascular diseases and weight in healthy working-age men while maintaining nutritional adequacy of iodine, iron, and vitamin B12 in the short term.

*Trial Registration* Clinical trial registration ClinicalTrials.gov as NCT04599920 in October 2020.

**Supplementary Information:**

The online version contains supplementary material available at 10.1007/s00394-025-03783-x.

## Introduction

The EAT-*Lancet* Commission advocates for the Planetary Health Diet, highlighting plant-source proteins over animal-source proteins to secure human and planetary health [1]. Decreasing red and processed meat (RPM) consumption is crucial for reducing both disease and environmental burden that arise from high RPM consumption [2, 3]. Legumes, which are low in saturated fat and high in protein and fiber, are recommended as a healthier, environmentally-friendly alternative to RPM [3, 4].

Increasing consumption of unprocessed red meat by 100 g and processed meat by 50 g has been associated with increased risk of colorectal cancer by 12% and 16%, respectively [2]. Reduction of 3 servings per week of either unprocessed red meat or processed meat has reduced risk of cardiovascular diseases (CVDs) by 5% and 3% and CVD mortality by 10%, respectively [5]. Conversely, increasing legume consumption by 100 g/d has been associated with a reduction of cancer risk by 21% and an increase of 50 g/d of legumes with 4% lower all-cause mortality [6]. In randomized controlled trials (RCTs), legume consumption of approximately 120–150 g/d reduced total and LDL cholesterol by 0.2 mmol/L, thus potentially protecting against CVDs [7, 8]. A meta-analysis of three long-term cohort studies confirmed a 16% decrease in coronary heart disease (CHD) incidence when RPM was replaced with legumes [9] while meta-analyses of RCTs reported approximately 0.2 mmol/L greater reductions of total and LDL cholesterol in favor of legumes and nuts compared with red meat [10] and similar result for LDL cholesterol when consuming 130 g/d of non-soy pulses compared with pulse-free isocaloric diets [11]. Observational studies and RCTs suggest that plant-rich diets support weight management more than animal-rich diets [12–14]. In RCTs, RPM consumption has not affected glycemic control in cardiometabolic disease-free individuals [15], whereas regular consumption of non-soy legumes has improved glycemic control in individuals with type 2 diabetes (T2D) [7].

Although a shift towards more plant-based diets is essential, especially in populations with high animal-source food consumption, potential nutritional drawbacks must be considered [16]. Compared to omnivorous diets, vegetarian and vegan diets have been associated with inadequate intake of critical micronutrients such as vitamin B12 and iodine, and iron in women [17]. In our previous 12-week RCT, a dose-dependent partial replacement of protein-containing animal food with protein-containing plant-based food decreased vitamin B12 and iodine intake and status without causing any changes in iron status [18]. A recent systematic review evaluating the impact of environmentally sustainable diets on micronutrients found reduced intakes of zinc, calcium, iodine, and vitamins B12, A, and D as likely consequences [19]. This review included 56 studies, but found only one RCT, demonstrating the acute need for carefully planned RCTs to study the causal impact of the dietary transition across diverse populations and culturally relevant diets.

In high-income societies including Nordic countries, a gender disparity in RPM consumption has been evident [20, 21]. For example, Finnish men consumed, on average, twice as much as women (762 vs. 392 g/wk) [22], which is double the upper limit of the Nordic Nutrition Recommendations (NNR) (350 g/wk) [4] and nearly four times the upper limit of the Planetary Health Diet (196 g/wk) [1]. Legume consumption, in turn, remains low in many Western food cultures, being, for instance, 1–14 g/d in Nordic populations [21].

We conducted an RCT to study the impact of a partial substitution of RPM with non-soy legumes, limiting RPM consumption to the maximum of the Planetary Health Diet. Faba bean and pea were favored as they can be cultivated in the Nordic conditions. The study targeted men, a group lagging behind in the transition to more sustainable diets [23, 24]. We analyzed the effects on macronutrient and critical micronutrient (iodine, iron, and vitamin B12) intakes and status, anthropometric measurements, and biomarkers of CVD and T2D risk in apparently healthy working-age men.

## Materials and methods

### Trial design and participants

**Fig. 1:**
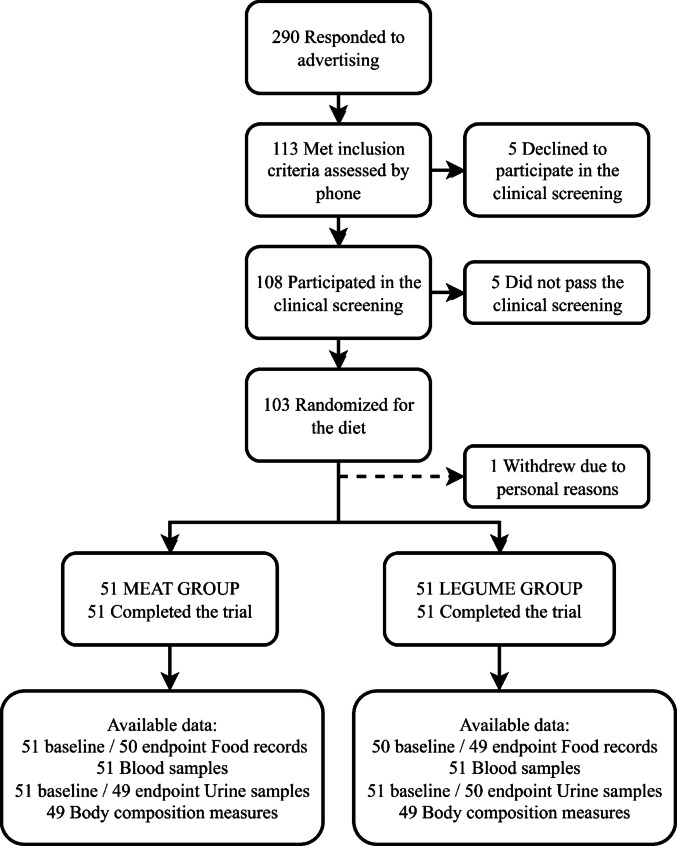
Recruitment and randomization of healthy men participating in the BeanMan 6-week RCT, where the diets of intervention groups differed in proportions of RPM and legumes. The MEAT group consumed 760 g/wk of RPM (25% of the TPI). The LEGUME group consumed legume-based products containing protein equivalent to 560 g/wk of red meat plus 200 g/wk of RPM; legumes accounted for 20% and RPM 5% of the TPI. RPM, red and processed meat; TPI, total protein intake

The BeanMan study is a 6-week randomized controlled trial conducted at the Department of Food and Nutrition, University of Helsinki, Finland, as a part of the large multidisciplinary research project ‘Legumes for Sustainable Food System and Healthy Life’ (Leg4Life, www.leg4life.fi). Participants were recruited starting from the beginning of September 2020 using mailing lists, intranet and social media platforms of the University of Helsinki, intranet of the Natural Resources Institute Finland, as well as Leg4Life project’s website and social media platforms. The trial’s target group was omnivorous men between the ages of 20 and 65 years old. Exclusion criteria were inflammatory bowel disease, irritable bowel syndrome, coeliac disease, diabetes requiring medical treatment, disorders of the endocrine system or lipid metabolism, liver or kidney disease, cancer, eating disorder, statin medication, regular or recent (in the last three months) use of antibiotics, allergy to the ingredients of foods provided by the study, strenuous long-lasting exercise several times a week, smoking or snuff use, high-risk alcohol consumption i.e., ≥ 24 portions per week [25], or traveling abroad within 14 days before the research visits that was related to restrictions during the COVID-19 pandemic. Of the 290 men interested in attending the study, 113 met the inclusion criteria based on a telephone interview, and were then invited to a screening visit (Fig. 1). During the screening visit, fingertip blood samples were collected to analyze total cholesterol and glucose after a 10–12 h overnight fast, and weight and height were measured for BMI calculation. Of the 108 men who attended the clinical screening, 103 passed the eligibility criteria of BMI within the range of 18.5 and 35.0 kg/m^2^, blood total cholesterol < 6.5 mmol/L, and fasting glucose < 7.0 mmol/L, and were therefore invited to participate in the intervention. The adequate number of participants was reached by mid-October 2020. As one participant withdrew from the study at this point, the final number of participants was 102. The time interval between the clinical screening and the beginning of the intervention was 5 days at minimum, but mostly 1–2 weeks.

The BuddyCare mobile application (version 4.39.0, Buddy Healthcare Ltd.) was adapted for this study to collect background data, including education level, alcohol consumption, leisure-time physical activity, work in terms of physical demand, and use of dietary supplements or medication. Due to the prevailing COVID-19 pandemic, study protocols were adapted to minimize the number and duration of face-to-face contacts between the researchers and participants. For example, the forms originally planned to be filled in during the research visits were instead filled in through the mobile application.

The BeanMan study was registered in ClinicalTrials.gov with the trial number NCT04599920, approved by the Coordinating Ethics Committee of the Hospital District of Helsinki and Uusimaa (HUS/1789/2020), and conducted according to the ethical principles of the Declaration of Helsinki. All participants were adult volunteers who provided a written informed consent and had the right to withdraw from the study at any time without explanation.

### Trial groups

The research coordinator generated the allocation sequence and randomized the participants to two equal-sized parallel diet groups adjusted for age. Blinding was not possible, but the participants were informed of their diet group’s color code. The intervention was implemented in a staggered manner, such that participants started their 6-week intervention during six consecutive weeks between mid-September and October 2020. In both diet groups, an equal number of participants started the trial each week.

During the intervention participants followed their habitual diets except for RPM and legumes. They were given either RPM (MEAT group) or a smaller amount of RPM plus legume-based foods (LEGUME group). The diet for the MEAT group was designed based on the Finnish national dietary survey, where total protein intake (TPI) was on average 98 g per day, representing 18% of total energy intake, and RPM accounted for one-fourth of the TPI in men [22]. Consequently, the MEAT group was given 760 g/wk of RPM (cooked and boneless weight), reflecting both the average consumption and quality of RPM in Finnish men, and corresponding to 25% of their TPI [22]. Similarly, to cover 25% of TPI, the LEGUME group was given 200 g/wk of RPM, providing 5% of TPI, and with non-soy legume-based foods (total product gross weight 940–1235 g/wk), providing the remaining 20% of TPI. In the LEGUME group, the amount of RPM was set close to the maximum amount in the Planetary Health Diet, i.e., 196 g/wk [1]. The sources of RPM included beef and pork, with products as raw meat, cold cuts, and sausages. Legume products were based on mainly pea and faba bean, including vegetable patties, products replacing minced meat or meat strips in cooking, pea soup, and frozen green peas. Products were non-fortified, although some included iodine-fortified salt. The participants were advised not to consume any additional RPM or legumes. They were asked to quit the use of dietary supplements and herbal medicinal products two weeks prior to the intervention.

The intervention foods were provided to the participants for free, and they received the foods weekly through home/workplace delivery or picked them up at the research center. Also, recipes were given to the participants to facilitate implementation of the diet. The food products were to be consumed within the specified time frame, and the participants submitted a weekly compliance diary on food consumption through the BuddyCare mobile application. Based on the compliance diaries, we calculated the amount of foodstuffs that were left unconsumed of the foods supplied by the trial. Also, compliance was verified by comparing the share of RPM and legumes of TPI based on food records with designed shares of TPI.

### Food records

Data on food consumption were collected on paper forms or in Excel at baseline and during the last week of the intervention using 4-day food records, including three consecutive weekdays and one weekend day. The baseline food records from one participant (1%) and the endpoint food records from three participants (3%) were not available for analyses of food consumption and nutrient intakes. At baseline, 4-day food record coverage was achieved by all participants and by 95 participants (96%) at the endpoint (Table 1).

**Table 1:** Characteristics of healthy men participating in the BeanMan 6-week RCT, categorized by the intervention group^a^

	MEAT	LEGUME	All
Characteristic	(*n* = 51)	(*n* = 51)	(*n* = 102)
Age, years, mean (SD)	38.9 (10.5)	36.9 (9.7)	37.9 (10.1)
Classified age, years, *n* (%)			
21–30	15 (29.4)	15 (29.4)	30 (29.4)
31–40	17 (33.3)	20 (39.2)	37 (36.3)
41–50	9 (17.6)	10 (19.6)	19 (18.6)
51–61	10 (19.6)	6 (11.8)	16 (15.7)
BMI, kg/m^2^, baseline, mean (SD)	25.5 (3.5)	25.6 (3.1)	25.6 (3.3)
Education level, *n* (%)			
High school or vocational qualification	8 (15.7)	14 (27.5)	22 (21.6)
Degree from a university of applied sciences or equivalent	12 (23.5)	10 (19.6)	22 (21.6)
Academic degree	31 (60.8)	27 (52.9)	58 (56.9)
Leisure-time exercise^b^, times/wk, n (%)			
not every week	7 (13.7)	4 (7.8)	11 (10.8)
1–2	12 (23.5)	10 (19.6)	22 (21.6)
3	19 (37.3)	17 (33.3)	36 (35.3)
4 or more	13 (25.5)	20 (39.2)	33 (32.4)
Type of work, n (%)			
Sedentary	38 (74.5)	36 (70.6)	74 (72.5)
Mobile or manual labor	10 (19.6)	13 (25.5)	23 (22.5)
Not working	3 (5.9)	2 (3.9)	5 (4.9)
Food record days^c^, baseline/endpoint, *n* (%)			
2	– / –	– / 1 (2.0)	– / 1 (1.0)
3	– / –	– / 3 (6.1)	– / 3 (3.0)
4	50 (98.0) / 50 (100.0)	50 (100.0) / 45 (91.8)	100 (99.0) / 95 (96.0)
5	1 (2.0) / –	– / –	1 (1.0) / –

^a^The MEAT group consumed 760 g/wk of RPM (25% of the TPI). The LEGUME group consumed legume-based products containing protein equivalent to 560 g/wk of red meat plus 200 g/wk of RPM; legumes accounted for 20% and RPM 5% of the TPI. RPM, red and processed meat; TPI, total protein intake

^b^An activity lasting at least half an hour and involving at least slight panting and sweating but not including commuting exercise

^c^MEAT group endpoint *n* = 50, LEGUME group baseline/endpoint *n* = 50/49

Video instructions were given to fill in food records and estimate portion sizes using weighing, package labels, or household measures. Written instructions were also provided, as well as an online portion size estimation booklet [26]. Nutritionists reviewed food records and contacted participants in case of any missing information or unclear recordings. Food records were manually entered by the nutritionists into the Aromi Diet software (version 14.10.2, CGI Inc.) where nutrient intakes were calculated utilizing both the Finnish national food composition database (Fineli, release 20, 27.6.2019, Finnish Institute for Health and Welfare, https://fineli.fi/fineli/en), and the Synkka product information service (GS1 Finland Oy). In case the participant had consumed a food product or a dish not included in the database, a new food item was created manually in the database. Food items were assigned to a food group and a single retention factor (EuroFIR) [27] per nutrient per food group was applied for calculating intakes of vitamin B12, total iron, and vitamin C by RStudio version 4·1·0. (R Core Team). To determine ingredient sources for nutrients, all composite dishes were disaggregated into ingredients, which were classified into 17 ingredient groups based on the classification of the Fineli database, with some modifications (Supplementary Table 1). Each ingredient group was classified as either plant-source or animal-source of nutrients, enabling calculation of intakes of protein and iron from these categories. Certain food items such as dairy products, industrial bread, and ready-made meals were not disaggregated into ingredients, but were categorized by full weight into one ingredient group according to the ingredient holding the greatest share in the product. Iodized salt was disaggregated from composite dishes, but not from all industrial bakery products or other foods fortified with iodized salt.

### Biochemical samples

Venous blood samples were collected after a 10–12 h overnight fast at baseline and endpoint. Plasma samples were analyzed for cholesterol (total, HDL, and LDL), triglycerides, glucose, and ferritin, as well as transferrin receptor (TfR) and serum samples for transcobalamin bound vitamin B12 (holoTC), C-peptide, and insulin and blood samples for hemoglobin by accredited methods (Helsinki University Hospital Laboratories, Finland; Supplementary Table 2). Homeostasis model assessment insulin resistance (HOMA1-IR = fasting insulin * fasting glucose / 22.5), and beta cell function (HOMA1-B = (fasting insulin * 20) / (fasting glucose—3.5)) were estimated from fasting glucose and insulin values, respectively. Furthermore, the participants collected 24-h urine samples at home at baseline and endpoint. They were provided with a collection jug and a cold storage box and instructed to start the collection in the morning after the first urine and stop collection with the first urine in the next morning included. In case any urine was wasted, they needed to report the estimated amount. Using urea concentration analyzed at the Department of Food and Nutrition, University of Helsinki, Finland, urinary volume and collection time, 24-h urea excretion was calculated to compute nitrogen excretion, which was needed to estimate protein excretion using Maroni’s formula [28]: protein excretion (g/d) = 6.25 * (nitrogen excretion (g/d) + 0.031 * body weight in kg). Using urinary iodine concentration analyzed by the accredited method (Vita Laboratories, Finland), urinary volume and collection time, urinary iodine (U-I) as 24-h excretion was calculated. For three participants (MEAT *n* = 2, LEGUME *n* = 1), urinary volume and collection time were not available at the endpoint and were excluded from the analysis.

### Anthropometric and body composition measurements

Height, waist, and hip circumferences were measured according to the instructions of the European Health Examination Survey Manual [29]. Height was measured using a stadiometer, and waist and hip circumferences using an inelastic measuring tape (Seca 201). Weight was measured using a digital personal scale (Seca 878) to the nearest 50 g. Body composition including relative fat mass as a percentage of body weight, absolute fat mass in kg, fat mass index (FMI) as fat mass in relation to height (kg/m^2^), absolute fat free mass in kg and fat free mass index (FFMI) as fat free mass in relation to height (kg/m^2^) were analyzed after a 10–12 h fast using the bioimpedance-based medical body composition analyzer (Seca mBCA 515). Body composition data were missing from four participants. All measurements, except height, were taken both at baseline and endpoint.

### Statistical analyses

The sample size was determined to show the effects of the trial on the serum concentration of holoTC and the concentration of N-nitroso compounds in feces, since they were expected to be the variables with the largest variation. The power calculation was based on the data of our previous dietary trials regarding the concentration of serum holoTC and faecal N-nitroso compounds [18, 30, 31], demonstrating that 50 participants per diet group were needed to detect statistical differences between groups at the end of the intervention with a 95% confidence interval and statistical power of 0.80.

Daily mean nutrient intakes were calculated, as well as proportions for each food ingredient group as sources of nutrients. An independent samples *t*-test was used to analyze differences between the diet groups in all nutrients at the endpoint. For other variables, differences between the diet groups at the endpoint were analyzed using analysis of covariance (ANCOVA), where the baseline value was used as a covariate. A paired samples *t*-test was used to analyze differences within the diet groups between baseline and endpoint. Spearman correlation was used to analyze the relationship between nutrient intake measured by food records and corresponding nutritional status biomarker.

Data were assessed for normality using the Kolmogorov–Smirnov test and were log10-transformed to enhance normality when required. In all tests, differences were considered significant at *P* < 0.05, but for the Spearman correlation, significance level was 0.01 (2-tailed). Statistical analyses were performed using IBM SPSS Statistics versions 28 and 29.

## Results

### Participants

The mean ± SD age of participants was 38 ± 10 years with a range of 21–61 years (Table 1). More than half of the participants had academic education and almost three quarters had sedentary-type work. Two-thirds reported undertaking leisure-time physical activity at least three times a week. Baseline BMI was similar in both groups (MEAT 25.5 ± 3.5 vs. LEGUME 25.6 ± 3.1 kg/m^2^) (Supplementary Table 3).

### Food consumption and compliance

The share of RPM of TPI was 26 ± 11% (designed 25%) in the MEAT and 5 ± 4% (designed 5%) in the LEGUME group; the share of legumes of TPI was 23 ± 9% (designed 20%) in the LEGUME group (Supplementary Table 4). The proportion of foodstuffs left unconsumed was 1–2% of the total amount of foods distributed to the participants. At the endpoint, consumption of legumes was 163 ± 50 g/d and that of RPM 26 ± 24 g/d in the LEGUME group (Table 2). In the MEAT group, RPM consumption was 148 ± 56 g/d and legume consumption 2.2 ± 5.7 g/d.

**Table 2:** Amount of food consumed by the MEAT and LEGUME groups based on 4-day food records at baseline and endpoint of the BeanMan 6-week RCT and change between endpoint and baseline

Ingredient group	MEAT (*n* = 51)	LEGUME (*n* = 50)	MEAT (*n* = 50)	LEGUME (*n* = 49)	MEAT (*n* = 50)	LEGUME (*n* = 49)
	Baseline (week 0)		Endpoint (week 6)		Change (Endpoint—Baseline) Mean (SD), g	
	Mean (SD), g		Mean (SD), g			
Vegetables	257 (124)	289 (131)	249 (151)	242 (150)	− 7.5 (132)	− 51.7 (127)
Nuts and seeds	14.0 (24.8)	17.6 (23.5)	12.0 (22.1)	15.9 (20.5)	− 2.3 (19.1)	− 2.1 (23.2)
Legumes	22.3 (34.2)	40.1 (47.4)	2.2 (5.7)	163 (49.6)	− 20.5 (34.3)	122 (66.0)
Pea and faba bean	9.7 (24.0)	15.5 (29.0)	0.3 (1.4)	154 (48.5)	− 9.6 (24.3)	138 (59.0)
Soy and other legumes	12.6 (23.6)	24.7 (32.2)	1.9 (5.1)	9.8 (17.5)	− 10.9 (24.4)	− 15.4 (32.9)
Potatoes	74.1 (77.3)	75.6 (60.4)	101 (89.9)	104 (85.0)	25.9 (98.2)	27.8 (86.7)
Fruits and berries	198 (195)	180 (155)	187 (174)	170 (158)	− 14.4 (126)	− 14.1 (146)
Cereals	270 (94.2)	296 (107)	250 (98.1)	265 (111)	− 18.6 (119)	− 21.8 (109)
Fat spreads, oils, and other fats	45.7 (19.9)	50.1 (22.5)	43.0 (20.1)	56.3 (24.1)	− 3.0 (19.7)	5.4 (30.4)
Fish and seafood	38.0 (44.6)	45.2 (46.9)	48.8 (55.1)	39.3 (40.4)	11.9 (71.3)	− 5.3 (49.8)
Red meat and processed red meat	90.5 (78.8)	90.1 (72.0)	148 (55.5)	26.4 (24.2)	59.7 (97.7)	− 61.5 (74.3)
Red meat	50.4 (60.8)	62.4 (72.2)	93.1 (45.1)	9.4 (20.6)	44.9 (73.6)	− 50.3 (67.8)
Processed red meat	40.1 (49.0)	27.7 (28.3)	55.2 (28.8)	17.0 (20.1)	14.8 (64.4)	− 11.2 (35.6)
White meat	73.9 (70.6)	66.1 (74.7)	53.9 (72.6)	42.6 (57.9)	− 20.9 (67.7)	− 21.2 (78.6)
Egg	26.3 (27.8)	25.6 (28.0)	27.6 (32.2)	25.2 (38.7)	1.0 (31.8)	− 0.9 (32.7)
Milk and dairy products	368 (292)	415 (268)	323 (228)	348 (250)	− 47.4 (171)	− 56.9 (156)
Plant-based dairy substitutes	61.1 (88.5)	74.4 (99.8)	52.9 (85.0)	79.3 (151)	− 9.4 (41.5)	3.4 (102)
Sugar, confectionery, and chocolate	38.8 (31.7)	39.5 (29.2)	39.4 (40.5)	39.0 (38.4)	0.6 (45.2)	− 1.2 (39.9)
Other products	34.8 (38.9)	36.8 (46.2)	29.5 (31.9)	21.7 (17.2)	− 5.3 (49.8)	− 15.5 (48.2)
Beverages	1857 (873)	2332 (959)	1856 (839)	2159 (845)	− 12.6 (690)	− 218 (496)
Alcohol	148 (220)	141 (182)	124 (191)	144 (216)	− 12.7 (179)	0.2 (193)

### Effects on macronutrient intakes

**Fig. 2:**
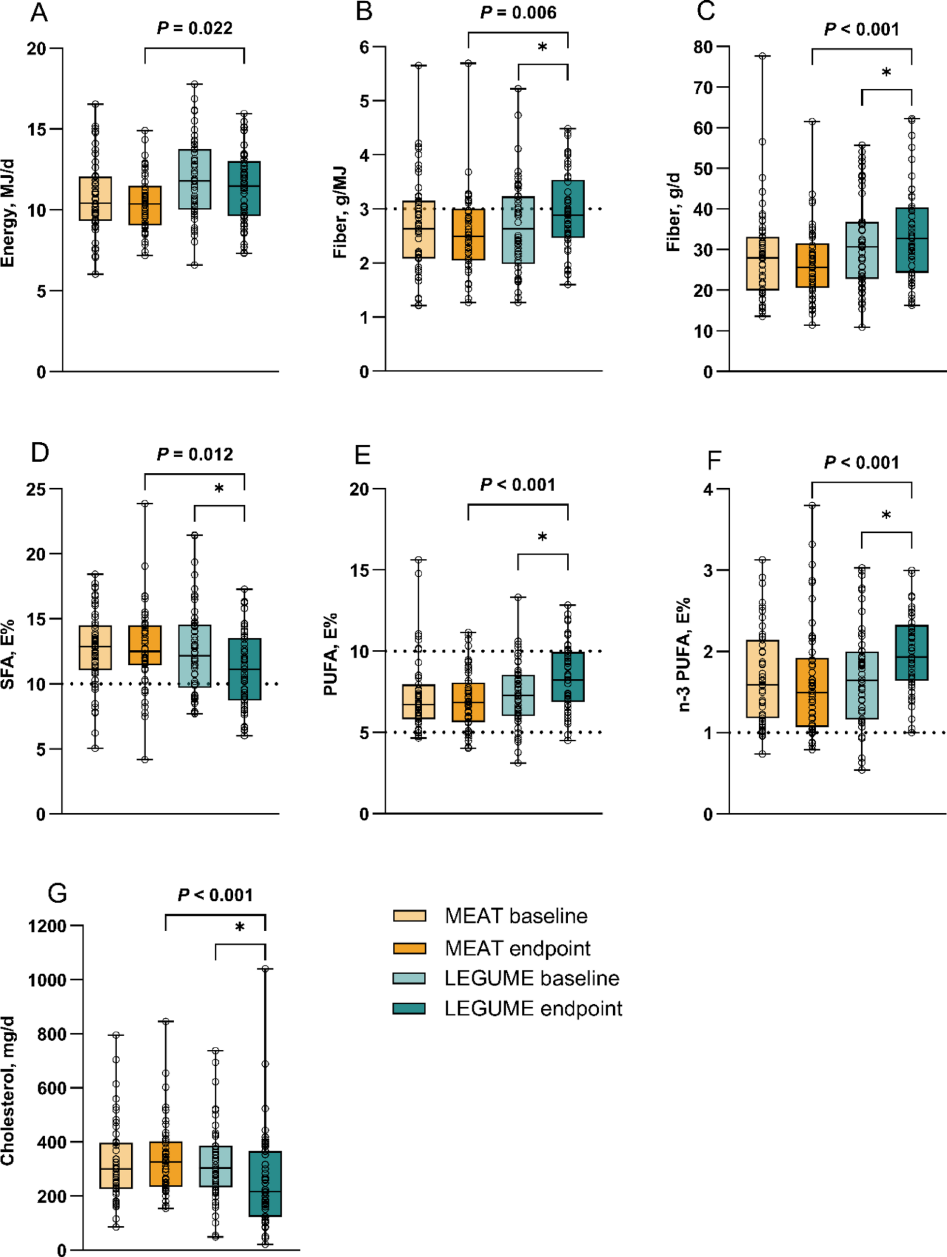
Daily mean intakes of **A** energy (MJ), **B** fiber (g/MJ), **C** fiber (g), **D** saturated fatty acids (SFA) (E%), **E** polyunsaturated fatty acids (PUFA) (E%), **F** n-3 PUFA (E%), and **G** cholesterol (mg) at baseline and endpoint of the BeanMan 6-week RCT. The MEAT group (baseline *n* = 51, endpoint *n* = 50) consumed 760 g/wk of RPM (25% of the TPI). The LEGUME group (baseline *n* = 50, endpoint *n* = 49) consumed legume-based products containing protein equivalent to 560 g/wk of red meat plus 200 g/wk of RPM; legumes accounted for 20% and RPM 5% of the TPI. Differences between the diet groups were analyzed by independent samples *t*-test. Asterisk (*) indicates a within-group difference between baseline and endpoint (P<0.05 at paired samples *t*-test). Boxplots represent the 25th percentile, median, 75th percentile, and whiskers (min to max) with individual values. Dotted line represents men’s minimum recommended intake (RI) for fiber, upper limit for SFA, range for PUFA, and minimum for n-3 PUFA [4]. E%, percentage of energy; RPM, red and processed meat; TPI, total protein intake

At the endpoint, energy intake was 1 MJ greater in the LEGUME than in the MEAT group (*P* = 0.022) (Fig. 2; Supplementary Table 5). No differences were found in protein intake (E%) or protein urinary excretion between the groups. On average, energy intake from protein was 16.9 ± 2.9 E% in the LEGUME group and 17.5 ± 2.8 E% in the MEAT group. Plant-source protein intake of TPI was 34% and 58% in the MEAT and LEGUME groups, respectively. Protein intakes analyzed by food records and urinary excretions correlated in both groups (MEAT *r* = 0.463, *P* < 0.001; LEGUME *r* = 0.588, *P* < 0.001).

Carbohydrate intake (E%) did not differ between the groups at the endpoint, but replacing RPM partly with legumes resulted in higher fiber intake both in grams (*P* < 0.001) and in g/MJ (*P* = 0.006). For fat intake (E%), no difference was found between the groups. Intakes of total polyunsaturated fatty acids (PUFAs), n-3 PUFAs, n-6 PUFAs, and α-linolenic acids (E%) were higher (all *P* < 0.001), while intakes of saturated fatty acids (SFA) in E% (*P* = 0.012) and cholesterol in mg (*P* < 0.001) were lower in the LEGUME than in the MEAT group.

### Effects on vitamin B12, iodine, and iron intake and status

**Fig. 3:**
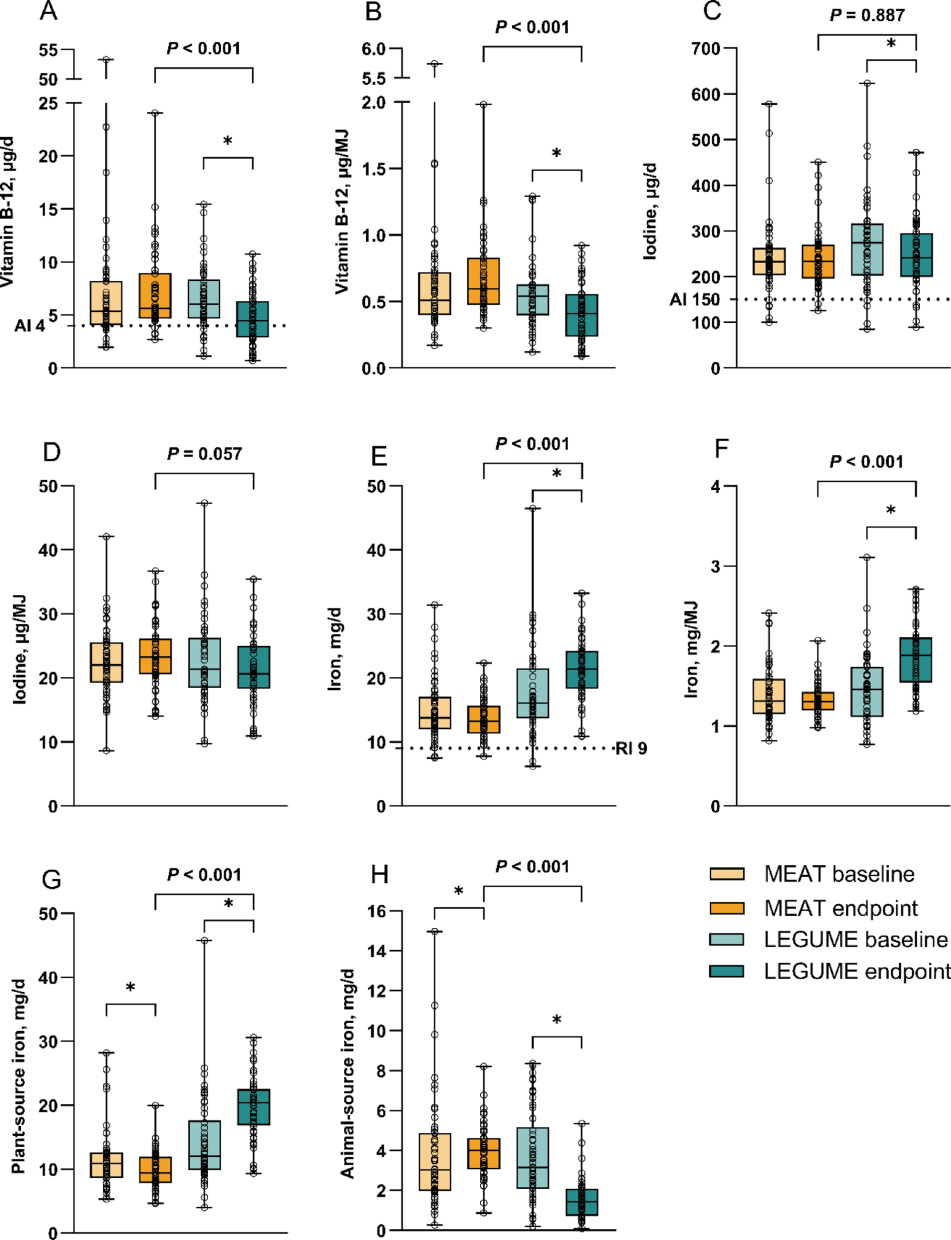
Daily mean intakes of **A** vitamin B12 (µg)^a^, **B** vitamin B12 (µg/MJ), **C** iodine (µg), **D** iodine (µg/MJ), **E** iron (mg), **F** iron (mg/MJ), **G** plant-source iron (mg), and **H** animal-source iron (mg) at baseline and endpoint of the BeanMan 6-week RCT. The MEAT group (baseline *n* = 51, endpoint *n* = 50) consumed 760 g/wk of RPM (25% of the TPI). The LEGUME group (baseline *n* = 50, endpoint *n* = 49) consumed legume-based products containing protein equivalent to 560 g/wk of red meat plus 200 g/wk of RPM; legumes accounted for 20% and RPM 5% of the TPI. Differences between the diet groups were analyzed by independent samples *t*-test. Asterisk (*) indicates a within-group difference between baseline and endpoint (P<0.05 at paired samples *t*-test). Ingredient groups for plant-source iron: vegetables, nuts and seeds, pea and faba bean, soy and other legumes, potatoes, fruits and berries, cereals, plant-based dairy substitutes, sugar, confectionery, and chocolate, other beverages; for animal-source protein: fish and seafood, red meat, processed red meat, white meat, egg, milk and dairy products. Boxplots represent 25th percentile, median, 75th percentile, and whiskers (min to max) with individual values. Dotted line represents men’s recommended intake (RI) for iron and adequate intake (AI) for iodine and vitamin B12 [4]. RPM, red and processed meat; TPI, total protein intake

**Fig. 4:**
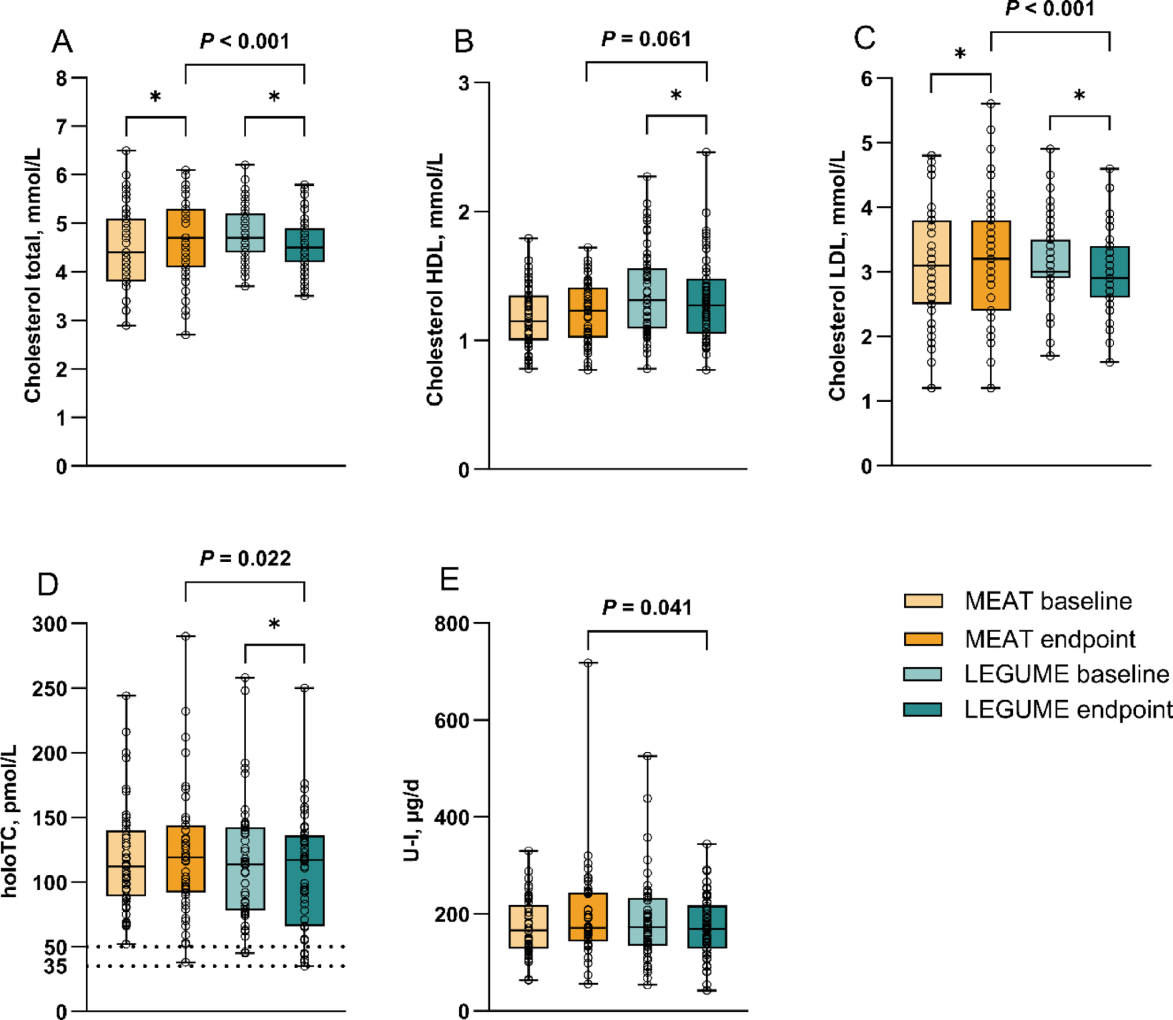
Biomarkers: **A** total cholesterol, **B** HDL cholesterol, **C** LDL cholesterol, **D** transcobalamin bound vitamin B12 (holoTC), and **E** urinary iodine (U-I) as 24-h excretion at baseline and endpoint of the BeanMan 6-week RCT. The MEAT group (*n* = 51 except for U-I *n* = 49) consumed 760 g/wk of RPM (25% of the TPI). The LEGUME group (*n* = 51 except for holoTC and U-I *n* = 50) consumed legume-based products containing protein equivalent to 560 g/wk of red meat plus 200 g/wk of RPM; legumes accounted for 20% and RPM 5% of the TPI. Boxplots represent 25th percentile, median, 75th percentile, and whiskers (min to max) with individual values. *P* from ANCOVA adjusted for baseline with Bonferroni correction. Asterisk (*) indicates a within-group difference between baseline and endpoint (*P* < 0.05 at paired samples *t*-test). For holoTC, the line at 35 pmol/L represents deficiency and at 50 pmol/L marginal status [32]. RPM, red and processed meat; TPI, total protein intake

At the endpoint, vitamin B12 intake was lower in the LEGUME group than in the MEAT group (4.7 ± 2.5 vs. 7.1 ± 3.8 µg/d, *P* < 0.001), and the same was true for vitamin B12 intake per MJ (*P* < 0.001) (Fig. 3; Supplementary Fig. 1; Supplementary Table 6). The concentration of vitamin B12 status indicator serum holoTC was also lower in the LEGUME group than in the MEAT group (*P* = 0.022), and this dropped within the LEGUME group (*P* = 0.022) during the trial (Fig. 4; Supplementary Table 3). One (2%) participant in the MEAT and eight (16%) in the LEGUME group had holoTC concentration below marginal status of 50 pmol/L [32] at the endpoint (Supplementary Fig. 2). This was also seen in two (4%) participants in the LEGUME group at baseline. Intake and status of vitamin B12 were correlated (*r* = 0.261, *P* = 0.009) in the entire study population at the endpoint.

Iodine intake did not differ between groups (MEAT 245 ± 71 vs. LEGUME 246 ± 76 µg/d) neither intake per MJ at the endpoint (Fig. 3; Supplementary Table 6). Iodine status biomarker U-I excretion was lower in the LEGUME group compared to the MEAT group (*P* = 0.041) (Fig. 4; Supplementary Table 3). Three (6%) participants in the MEAT and seven (14%) in the LEGUME group had endpoint U-I excretion below 100 µg/d, which is the bottom level considered for adequate iodine intake [33, 34]. At baseline, the same applied for two (4%) participants in the MEAT and seven (14%) in the LEGUME group. Intake and status of iodine were correlated (*r* = 0.503, *P* < 0.001) in the entire study population at the endpoint.

The partial substitution of RPM with legumes led to higher intake of total iron in mg (21 ± 5 vs. 14 ± 3 mg/d) and per MJ, as well as higher intake of plant-source iron but lower intake of animal-source iron (all *P* < 0.001) (Fig. 3; Supplementary Table 6). The proportion of plant-source iron from total intake was 69% in the MEAT group and 90% in the LEGUME group. The ratio of plant-source iron to animal-source iron was 7.5-fold in the LEGUME (23 ± 35 mg/d) compared with the MEAT group (3.0 ± 2.3 mg/d). Within a group decrease in molar ratio of vitamin C to iron in the LEGUME group was seen between baseline and endpoint (*P* = 0.028). No significant differences in iron status indicators (plasma ferritin, plasma TfR, and hemoglobin) were found between the diet groups (Supplementary Table 3). In the LEGUME group, ferritin declined during the trial (*P* < 0.001).

### Food sources of nutrients

In the MEAT group, the main protein sources were RPM (26% of TPI), cereals (20%), and milk and dairy products (20%). In the LEGUME group, legumes accounted for 23% of TPI, followed by milk and dairy products (22%), and cereals (21%) (Supplementary Table 4). Cereals provided the greatest share of fiber in both groups (MEAT 53%, LEGUME 43%). Legumes accounted for one-fifth of fiber intake in the LEGUME group. RPM (12% of total intake) in the MEAT group, and legumes (14%) in the LEGUME group were the third largest source of fat, the former contributing to SFA intake and the latter to PUFA intake, after fat spreads, oils, and other fats and milk and dairy products (Supplementary Tables 7 and 8).

**Fig. 5:**
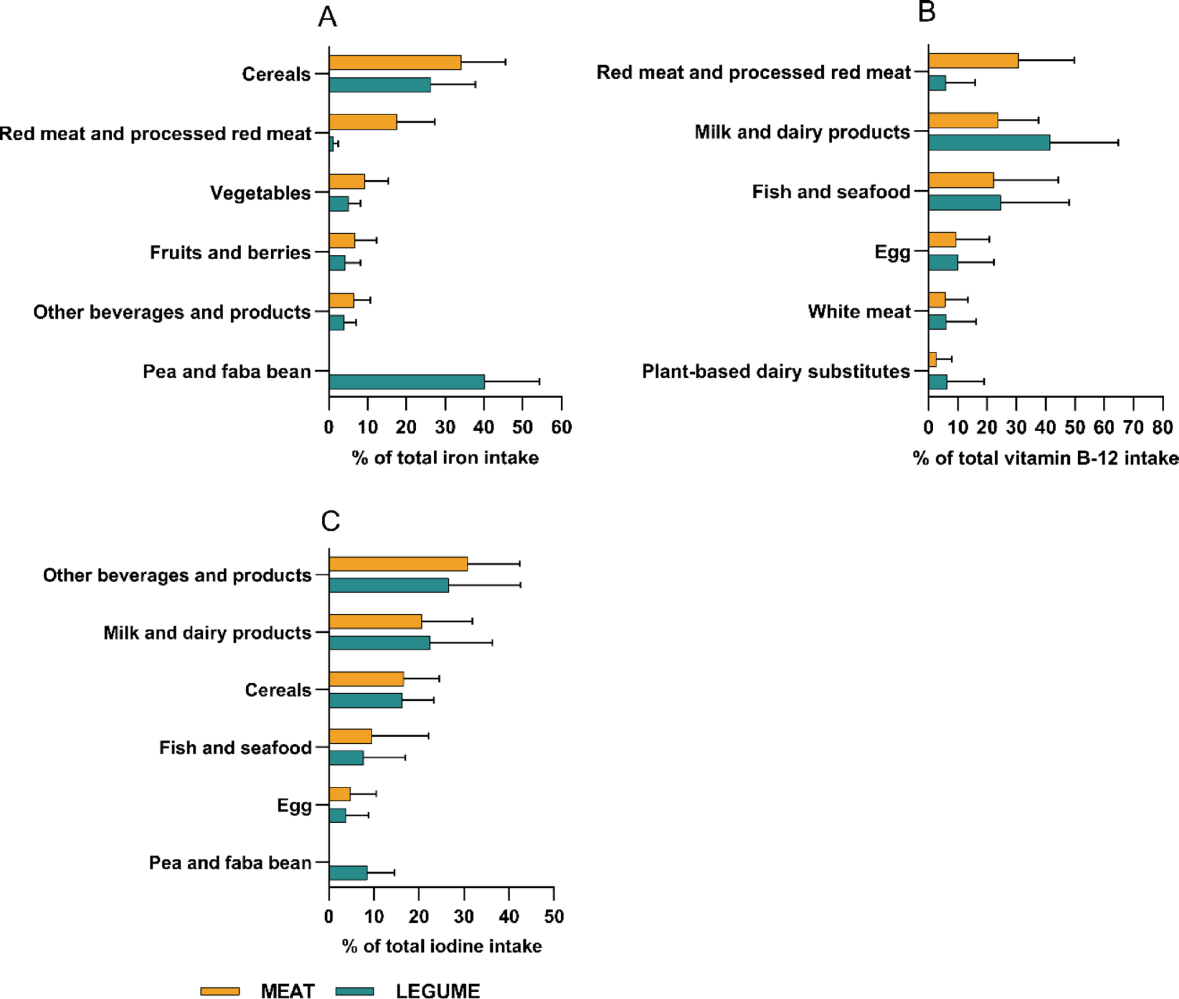
Main sources of **A** iron, **B** vitamin B12, and **C** iodine (other beverages and products includes iodized salt) as daily average proportions with SD from the ingredient groups at the endpoint of the BeanMan 6-week RCT. The MEAT group (*n* = 50) consumed 760 g/wk of RPM (25% of the TPI). The LEGUME group (*n* = 49) consumed legume-based products containing protein equivalent to 560 g/wk of red meat plus 200 g/wk of RPM; legumes accounted for 20% and RPM 5% of the TPI. RPM, red and processed meat; TPI, total protein intake

In the MEAT group, the main sources of vitamin B12 were RPM (31% of total intake) and milk and dairy products (24%), while in the LEGUME group, milk and dairy products accounted for 42% of the total intake (Fig. 5; Supplementary Table 9). Fish and seafood contributed to vitamin B12 intake by 22% and 25% in the MEAT and LEGUME groups, respectively. The proportion of iodine intake from iodized salt was 27% in the MEAT group, and 22% in the LEGUME group. Besides iodized salt, the main sources of iodine were milk and dairy products and cereal products in both groups. Of the total iron intake, the share of legumes was 42% in the LEGUME group, and the share of RPM was 18% in the MEAT group. Cereals were the most important source of iron in the MEAT group, contributing 34% of total intake, and the second most important (26%) in the LEGUME group.

### Effects on anthropometric parameters, body composition, and blood biomarkers

At the endpoint, weight and BMI (25.3 ± 3.1 vs. 25.5 ± 3.5 kg/m^2^) were lower in the LEGUME than in the MEAT group (*P* = 0.009) (Supplementary Table 3). During the intervention, weight loss was 1.0 ± 1.5 kg in the LEGUME group (*P* < 0.001) and 0.3 ± 1.2 kg in the MEAT group (*P* = 0.120). BMI decreased significantly in the LEGUME group (*P* < 0.001). No endpoint differences were seen in waist or hip circumference, or waist-hip ratio between the diet groups. Within the trial, hip circumference decreased in both groups (*P* < 0.001), but for waist circumference, the decrease was significant only in the LEGUME group (*P* = 0.003); for the waist-hip ratio, a significant increase was seen in the MEAT group (*P* = 0.013). In relative fat mass, absolute fat mass, fat mass index, fat free mass, or fat free mass index, no differences were observed between the groups. In the LEGUME group, all body composition measures decreased during the trial (*P* = 0.005–0.045). No between-group differences were observed in plasma glucose, serum C-peptide and insulin, HOMA1-IR, or HOMA1-B indices at the end of the trial, but C-peptide increased in both diet groups from baseline to endpoint (*P* = 0.001–0.002).

Partially substituting RPM with legumes resulted in lower total and LDL cholesterol concentrations (both *P* < 0.001), but no significant differences between the groups were found for HDL cholesterol or triglycerides (Fig. 4; Supplementary Table 3). During the trial, total cholesterol increased in the MEAT (0.14 ± 0.45 mmol/L, *P* = 0.036), but decreased in the LEGUME group (− 0.25 ± 0.41 mmol/L, *P* < 0.001). Moreover, LDL cholesterol increased in the MEAT (0.16 ± 0.52 mmol/L, *P* = 0.030), but decreased in the LEGUME group (− 0.19 ± 0.45 mmol/L, *P* = 0.004). In turn, HDL cholesterol did not change significantly in the MEAT (0.02 ± 0.14 mmol/L), but declined in the LEGUME group (− 0.05 ± 0.12 mmol/L, *P* = 0.004).

## Discussion

The transition towards more plant-rich diets requires more attention on the possible negative nutritional impacts [35]. In particular, well-executed trials to identify cause and effect relationships between sustainable diets and health are urgently needed. In this 6-week RCT, limiting RPM consumption to the maximum of the Planetary Health Diet (200 g/wk; 5% of TPI) alongside the use of non-soy legumes to replace RPM to cover 20% of TPI favorably changed the fatty acid composition of the diet and increased fiber and iron intakes in healthy working-age men. Furthermore, this dietary shift beneficially affected blood lipoprotein profile, weight, and BMI while maintaining adequate intake and status of iodine, iron, and vitamin B12.

Diets that include meat typically have higher protein intake in comparison to vegetarian diets [17]. In our study, protein intake was similar (around 17 E%) in both diet groups, complying well with the NNR target of 10–20 E% [4] and our trial design (18 E%). Also, intake of essential amino acids was adequate in both groups [36]. In non-meat diets, fiber intake is usually higher than in diets that include meat [17]. In the LEGUME group, fiber intake increased to 2.9 g/MJ during the intervention, nearly reaching the NNR target level of at least 3.0 g/MJ for men [4].

Compared to the MEAT group, higher unsaturated and lower saturated fatty acid intakes in the LEGUME group may explain lower concentrations of total and LDL cholesterol, with the average dose of 163 g/d of legumes mostly provided as convenience foods. Moreover, the results may be partly explained by leguminous soluble fiber [37] and saponins [38]. Similar to our study, the meta-analysis of 25 RCTs reported that consuming a portion of 130 g/d of whole non-soy pulses (beans, chickpeas, lentils, and peas) for six weeks led to a difference of −0.17 mmol/L in LDL cholesterol [11]. In another meta-analysis of eight RCTs, the consumption of legumes, soy, and other plant-source proteins resulted in more beneficial changes in total and LDL cholesterol compared with red meat consumption [10]. The meta-analysis of observational studies, in turn, found that replacing one serving (e.g. 1/2 cup) per day of red meat with the same amount of legumes was associated with lower CHD occurrence [9].

Interestingly, both the MEAT and LEGUME groups lost weight during the trial, but the weight loss and consequent reduction in BMI was significantly greater in the LEGUME than the MEAT group at the endpoint. A meta-analysis of prospective studies demonstrated that legume consumption is associated with lower but red meat consumption with higher risk of weight gain, overweight, and obesity [12]. Furthermore, meta-analyses of RCTs reported a greater weight loss potential of vegetarian diets compared with non-vegetarian diets [13, 14]. In our study, throughout the trial, the LEGUME group had one megajoule higher energy intake than the MEAT group, likely explained by a higher leisure-time exercise frequency in the LEGUME group. Moreover, weight loss in both groups could be due to a typical bias associated with dietary interventions, as participants may have altered their food consumption to show compliance with the objectives of the trial [39].

Despite the favorable changes in weight and BMI, we found no differences between the diet groups in the biomarkers of T2D risk. This could be because our study involved generally healthy non-obese individuals, aligning with the systematic review of RCTs where the protective effect of non-soy legume consumption on fasting blood glucose or insulin was found only in individuals with T2D [40]. Furthermore, in the meta-analysis of RCTs related to red meat intake, no effect on blood glucose and insulin or HOMA-IR index was observed in individuals free from cardiometabolic disease [15]. However, fasting serum C-peptide increased by 11% in both groups during the trial. While insulin and C-peptide are secreted in equimolar amounts, C-peptide is primarily cleared by the kidneys and insulin by the liver, respectively. It is possible that a small change in either clearance of insulin or C-peptide contributed to the observed difference. To test this was, however, beyond the scope of the study. 

In the LEGUME group, reduced intake of vitamin B12 was manifested as an 8% reduction in the serum holoTC, the most sensitive indicator of both recent vitamin B12 intake and vitamin B12 deficiency [32]. In the previous 12-week trial, we observed a dose-dependent decrease in vitamin B12 intake [18]; lower intakes reflected lower concentrations of serum holoTC, with the most significant reduction being 23% when plant-source proteins were mostly replaced with animal-source proteins. Moreover, our findings are in line with the prior 4-week RCT, where a decrease of both vitamin B12 intake and serum holoTC were significant in a non-supplemented vegan diet compared with diets including meat [41]. However, in the current 6-week trial with a reduction of RPM to around 200 g/wk vitamin B12 remained above the adequate intake of 4.0 µg/d suggested by the European Food Safety Authority [42] and the NNR [4], and holoTC at least 35 pmol/L (lower values indicating deficiency) [32]. In omnivorous diets, vitamin B12 is derived from animal-source foods [22]. Apart from the planned changes in RPM and legume consumption, our trial led to an over 10% lower consumption of milk and dairy products in both groups, while consumption of fish and seafood and plant-based dairy substitutes typically fortified in vitamin B12 were differently changed in the dietary groups. Thus, changes in the habitual diet may have also influenced micronutrient intakes. In this short-term study, a moderate dietary shift did not jeopardize vitamin B12 intake and status, but when reducing the relative share of animal-source foods permanently and significantly, vitamin B12 intake should be monitored and ensured by consuming fortified foods, especially in vulnerable population groups.

Previously, iodine intake and status have been lower in diets with reduced amounts of animal-source foods and plant-based diets [17, 18]. Similarly, in this study, iodine status was lower in the LEGUME group than in the MEAT group, but for iodine intake no difference was found. In both groups, iodine intake was well beyond the adequate intake of 150 µg/d [4], and U-I excretion was within the adequate range [33, 34]. In Finland, nutritional policy instruments have been used to improve the population’s iodine intake, and currently iodized salt is widely used in home cooking, food and bakery industry, and food services [43]. Consequently, iodized salt was the main source of iodine followed by milk and dairy products. Finally, it must be noted that the lack of retention factors most likely led to an overestimation of iodine intake in this study.

Baseline iron intake was close to double the recommended intake of 9 mg/d [4] and increased in the LEGUME but decreased in the MEAT group during the trial. The established iron status biomarkers, *i.e.*, ferritin, Tfr, and hemoglobin [44] did not differ between the groups after six weeks, possibly due to abundant baseline intake. Similarly, in the prior 12-week trial, we observed no significant difference in iron status between the diets with lower and higher shares of animal-source proteins, but iron intake was higher in the former group [18]. In the LEGUME group, a weekly intake of 200 g of RPM may have contained enough heme iron, which is highly bioavailable, to prevent significant depletion of iron stores. Furthermore, animal-source muscle tissue of white meat, fish, and RPM as well as vitamin C could have enhanced the absorption of plant-source iron by reducing the ferric iron (Fe^3+^) to its ferrous state (Fe^2+^) and forming absorbable chelates with iron [44], thus supporting the bioavailability of the non-heme iron in the LEGUME group. It is also recommended that for high-phytate foods such as legumes, a molar ratio of ascorbic acid to iron should be 2:1 or higher [44], which was reached in the LEGUME group even though the ratio was not increased during the trial as iron intake was increased proportionally more than vitamin C intake.

The strengths of this study include that no one dropped out, indicating that the diets were feasible to follow. Further strengths were comprehensive assessment of food and nutrient intakes, and well-established nutritional status measurements including a 24-h urine sample. In addition, participants’ compliance was good, since their consumption of legumes and RPM corresponded to the plan. However, the participants were highly educated and probably more health-conscious than an average citizen, which may limit the generalizability of the results. Health-consciousness was evident, for example, participants’ baseline fiber intake was ≥ 30% higher than on average in the population [22]. Hence, we suppose that the results would have been more pronounced if studied in the general population.

## Conclusion

We demonstrated that consuming non-soy legumes to substitute protein derived from RPM and limiting weekly RPM consumption to the maximum of the Planetary Health Diet for six weeks enhanced the fatty acid composition of the diet, fiber, and iron intakes, and positively impacted the blood lipoprotein profile, weight, and BMI among healthy working-age men. Consequently, a moderate dietary change towards a more sustainable direction, i.e., partly shifting from animal-source proteins to plant-source proteins, may result in beneficial effects on biomarkers of CVDs while maintaining adequate intake and status of critical micronutrients such as iodine, iron, and vitamin B12, at least in the short term. Our research suggests that from the perspectives of nutritional adequacy and acceptability, the maximum of 350 g/wk for red meat newly set by the NNR [4]—replacing the previous recommendation of 500 g/wk [45]—is realistic and achievable, even among men whose current consumption is twice that amount. In the future, more longer-term dietary trials are needed to investigate the impact of changes in relative shares of animal-source and plant-source foods, incorporating bioavailability of nutrients and involving different food cultures, and particularly in vulnerable population groups.

## Supplementary Information

Below is the link to the electronic supplementary material.

Supplementary Material 1

## Data Availability

Data described in the manuscript, code book, and analytic code will be made available upon request pending application and approval in agreement with the EU General Data Protection Regulation.

## Abbreviations

AI: Adequate intake
FFMI: Fat free mass index
FMI: Fat mass index
holoTC: Transcobalamin bound vitamin B12 (holotranscobalamin)
HOMA1-B: Homeostasis model assessment of beta cell function
Leg4Life: Legumes for sustainable food system and healthy life
LEGUME: A diet including legume-based products containing protein equivalent to 560 g/wk of red meat in addition to 200 g/wk of red and processed meat (beef and pork); legumes accounting for 20% and red and processed meat for 5% of the total protein intake
MEAT: A diet including 760 g/wk of red and processed meat (beef and pork) accounting for 25% of the total protein intake
NNR: Nordic Nutrition Recommendations
RPM: Red and processed meat
TPI: Total protein intake
U-I: Urinary iodine
