# Supplementary material for: Nutritional and health benefits of a partial substitution of red and processed meat with non-soy legumes: a 6-week randomized controlled trial in healthy working-age men
Source: Eur J Nutr. 2025 Aug 19;64(6):259. doi: 10.1007/s00394-025-03783-x (PMC12364987; doi:10.1007/s00394-025-03783-x)
Supplement: Supplementary file 1 — Supplementary Material 1 [file 394_2025_3783_MOESM1_ESM.docx]

**SUPPLEMENTARY MATERIAL**

**Journal:** *European Journal of Nutrition*

**Article title:** Nutritional and health benefits of a partial substitution of red and processed meat with non-soy legumes: a 6-week randomized controlled trial in healthy working-age men

**Authors:** Sari Bäck, Essi Päivärinta, Tiina Pellinen, Suvi T Itkonen, Mikko Lehtovirta, Maijaliisa Erkkola, Niina E Kaartinen, Satu Männistö, Anne-Maria Pajari

**Corresponding author:**

Anne-Maria Pajari, E-mail: anne-maria.pajari@helsinki.fi

University of Helsinki, Department of Food and Nutrition, Helsinki, Finland

**Contents:**

[Supplementary Table 1](#SupplementaryTable1)

[Supplementary Table 2](#SupplementaryTable2)

[Supplementary Table 3](#SupplementaryTable3)

[Supplementary Table 4](#SupplementaryTable4)

[Supplementary Table 5](#SupplementaryTable6)

[Supplementary Table 6](#SupplementaryTable7)

[Supplementary Table 7](#SupplementaryTable8)

[Supplementary Table 8](#SupplementaryTable9)

[Supplementary Table 9](#SupplementaryTable10)

[Supplementary Figure 1](#SupplementaryFigure1)

[Supplementary Figure 2](#SupplementaryFigure2)

**SUPPLEMENTARY TABLE 1** Ingredients and food items included in the ingredient groups of the BeanMan 6-week RCT

| Ingredient group | Ingredients, food items and ready-made meals^a^ |
| --- | --- |
| Vegetables | Root vegetables, tubers (except potato), leaf vegetables, fruit vegetables, fungi, other vegetables; Ready-made meal: chicken soup |
| Nuts and seeds | Nuts, seeds |
| Pea and faba bean | Pea, faba bean |
| Soy and other legumes | Soy, legumes (except pea and faba bean) |
| Potatoes | Potatoes; Ready-made meals: salmon soup, salmon potato casserole, chicken casserole |
| Fruits and berries | Citrus fruit, malaceous fruit, other fruits, berries |
| Cereals | Wheat, rye, oat, barley, rice, and other grains; Industrial bread; Ready-made meals: fast food hamburger, curry chicken with rice |
| Fat spreads, oils, and other fats | Oils, margarine, vegetable fat spreads, butter, fat blends, other fats |
| Fish and seafood | Fish, seafood; Ready-made meal: salmon pasta casserole |
| Red meat | Beef, pork, lamb, game, offal |
| Processed red meat | Sausages, frankfurters, meat products and cold cuts made from red meat |
| White meat | Chicken and turkey unprocessed meat; processed meat: sausages, frankfurters, other meat products and cold cuts made from chicken and turkey meat |
| Egg | Eggs |
| Milk and dairy products | Milks, sour milk products, cheese, other milk products; Ready-made meal: chicken pasta casserole |
| Plant-based dairy substitutes | Fermented plant-based products, vegetable fat-based blends, other plant-based products |
| Sugar, confectionery, and chocolate | Sugar, confectionery, and chocolate |
| Other products and beverages | Iodized salt, other food products and, other beverages including alcohol |

^a^Ready-made meals were categorized by full weight into one ingredient group based on the main ingredient. Only the most consumed ready-made meals are listed

**SUPPLEMENTARY TABLE 2** Details of biochemical analyses in the BeanMan 6-week RCT

| Analysis | Method | Analyzer | CV% intra-assay | CV% inter-assay |
| --- | --- | --- | --- | --- |
| Cholesterol, total, plasma | photometric | Siemens Atellica | 1.0 | 2.2 at low level  1.6 at high level |
| Cholesterol, HDL, plasma | photometric | Siemens Atellica | 0.9 | 2.9 |
| Cholesterol, LDL, plasma | photometric | Siemens Atellica | 1.3 | 2.7 |
| Triglycerides, plasma | photometric | Siemens Atellica | 0.7 | 2.2 at low level  1.7 at high level |
| Glucose, plasma | photometric | Siemens Atellica | 0.8 | 2.4 at low level  1.7 at high level |
| C-peptide, serum | immunochemiluminometric | Siemens Atellica IM1600 | 5 | 8 at level <0,5 nmol/L  5 at level >0.5 nmol/L |
| Insulin, serum | immunochemiluminometric | Diasorin Liaison XL | 1.5 | 4 |
| HoloTC, serum | immunochemiluminometric | Siemens Atellica IM1600 | 5 | 7 |
| U-I excretion | inductively coupled plasma mass spectrometry | Agilent 7700 ICP-MS | 1.7 | 8.1 |
| Hemoglobin, blood | photometric | Siemens Atellica | 1.0 | 4.4 at low level  1.6 at high level |
| Ferritin, plasma | immunochemiluminometric | Siemens Atellica IM1600 | 5 | 7 |
| TfR, plasma | photometric, immunochemical | Siemens Atellica CH930 | 4 | 7 |
| Urea, urine | photometric | Indiko automatic analyzer | <4.6 | <4.6 |

HDL, high-density lipoprotein; holoTC, transcobalamin bound vitamin B12 (holotranscobalamin); LDL, low-density lipoprotein; TfR, transferrin receptor; U–I, urinary iodine

**SUPPLEMENTARY TABLE 3** Anthropometric measures, body composition, and blood and urine biomarkers for MEAT and LEGUME groups^a^ in the BeanMan 6-week RCT

|  | MEAT (*n* = 51) | LEGUME (*n* = 51) | |  | | MEAT (*n* = 51) | | LEGUME (*n* = 51) | | *P* value between groups, endpoint^b^ |  |
| --- | --- | --- | --- | --- | --- | --- | --- | --- | --- | --- | --- |
|  | Baseline (week 0)  Mean (SD) | | |  |  | Endpoint (week 6)  Mean (SD) | | | |  |  |
|  |  |  |  |  |  |  |  |  |  |  |  |
| Anthropometric parameters |  |  |  | |  | |  | |  | | |
| Weight, kg | 84.5 (13.3) | 84.9 (12.6) |  | | 84.2 (13.3) | | 83.9 (12.6)^c^ | | **0.009**^d^ | | |
| BMI, kg/m^2^ | 25.5 (3.5) | 25.6 (3.1) |  | | 25.5 (3.5) | | 25.3 (3.1)^c^ | | **0.009**^d^ | | |
| Waist circumference, cm | 88.8 (10.2) | 88.1 (10.1) |  | | 88.6 (10.4) | | 87.2 (9.7)^c^ | | 0.091^d^ | | |
| Hip circumference, cm | 98.3 (6.5) | 99.4 (7.6) |  | | 97.0 (6.4)^c^ | | 97.9 (6.7)^c^ | | 0.965 | | |
| Waist-hip ratio | 0.90 (0.06) | 0.89 (0.05) |  | | 0.91 (0.06)^c^ | | 0.89 (0.05) | | 0.116 | | |
| Body composition^e^ |  |  |  | |  | |  | |  | | |
| Relative fat mass, % | 23.7 (6.0) | 21.7 (6.7) |  | | 23.7 (6.2) | | 21.3 (6.7)^c^ | | 0.120 | | |
| Absolute fat mass, kg | 20.7 (7.7) | 19.2 (8.6) |  | | 20.7 (8.1) | | 18.6 (8.5)^c^ | | 0.100^d^ | | |
| Fat mass index (FMI), kg/m^2^ | 6.3 (2.3) | 5.7 (2.5) |  | | 6.2 (2.4) | | 5.6 (2.4)^c^ | | 0.098^d^ | | |
| Fat free mass, kg | 64.4 (7.2) | 66.2 (6.4) |  | | 64.1 (7.0) | | 65.8 (6.3)^c^ | | 0.917^d^ | | |
| Fat free mass index (FFMI), kg/m^2^ | 19.5 (1.7) | 19.9 (1.5) |  | | 19.4 (1.7) | | 19.8 (1.5)^c^ | | 0.916 | | |
| Biomarkers |  |  |  | |  | |  | |  | | |
| Cholesterol total, plasma, mmol/L | 4.5 (0.8) | 4.8 (0.5) |  | | 4.7 (0.8)^c^ | | 4.5 (0.5)^c^ | | **<0.001** | | |
| Cholesterol HDL, plasma, mmol/L | 1.2 (0.3) | 1.4 (0.3) |  | | 1.2 (0.2) | | 1.3 (0.3)^c^ | | 0.061 | | |
| Cholesterol LDL, plasma, mmol/L | 3.1 (0.8) | 3.2 (0.6) |  | | 3.2 (0.9)^c^ | | 3.0 (0.6)^c^ | | **<0.001** | | |
| Triglycerides, plasma, mmol/L | 1.1 (0.5)^f^ | 1.0 (0.5) |  | | 1.1 (0.6)^f^ | | 1.0 (0.4) | | 0.561^d^ | | |
| Fasting glucose, plasma, mmol/L | 5.3 (0.4) | 5.3 (0.4) |  | | 5.3 (0.4)^f^ | | 5.2 (0.4) | | 0.323 | | |
| Fasting C-peptide, serum, nmol/L | 0.63 (0.23) | 0.56 (0.18) |  | | 0.70 (0.23)^c^ | | 0.62 (0.24)^c^ | | 0.534^d^ | | |
| Fasting insulin, serum, mU/ml | 8.7 (5.2) | 7.0 (4.1) |  | | 8.0 (4.3) | | 6.6 (4.2) | | 0.397^d^ | | |
| HOMA1-IR insulin resistance | 2.09 (1.35) | 1.67 (1.04) |  | | 1.90 (1.03)^f^ | | 1.56 (1.06) | | 0.308^d^ | | |
| HOMA1-B beta cell function | 94.5 (49.8) | 79.6 (42.6) |  | | 92.7 (56.9)^f^ | | 77.8 (42.3) | | 0.826^d^ | | |
| Biomarkers for nutritional status |  |  |  | |  | |  | |  | | |
| HoloTC, serum, pmol/L | 118 (39.9) | 116 (45.5)^g^ |  | | 120 (47.3) | | 107 (45.1)^c^ | | **0.022** | | |
| U-I excretion^h^, µg/d | 173 (59.3) | 187 (88.5) |  | | 196 (96.8) | | 174 (64.1) | | **0.041** | | |
| Hemoglobin, blood, g/L | 145 (9.8) | 146 (8.4) |  | | 144 (8.1)^f^ | | 145 (8.2) | | 0.290 | | |
| Ferritin, plasma, µg/L | 110 (78.5) | 100 (63.8) |  | | 106 (75.9) | | 90.3 (59.8)^c^ | | 0.201 | | |
| TfR, plasma, mg/L | 0.79 (0.26) | 0.73 (0.21) |  | | 0.74 (0.23) | | 0.73 (0.19) | | 0.506 | | |

^a^The MEAT group followed a diet including 760 g/wk (cooked weight) of red and processed meat (beef and pork), accounting for 25% of the total protein intake. The LEGUME group followed a diet including legume-based products containing protein equivalent to 560 g/wk (cooked weight) of red meat, plus 200 g/wk of red and processed meat (beef and pork); legumes accounted for 20% and red and processed meat 5% of the total protein intake

^b^*P* from ANCOVA adjusted for baseline with Bonferroni correction. Bold means that the *P* is <0.05

^c^Statistically significant (two-sided *P* <0.05) difference within a group between baseline and endpoint according to paired samples *t*-test

^d^For log10-transformed variables

^e^MEAT group *n* = 49, LEGUME group *n* = 49. No proper data were collected from 4 participants

^f^MEAT group *n* = 50. From triglycerides data, 1 outlier was removed; for glucose and HOMA1 analyses, 1 sample was missing

^g^LEGUME group *n* = 50

^h^MEAT group *n* = 49, LEGUME group *n* = 50. No proper samples were received from 3 participants

HDL, high-density lipoprotein; holoTC, transcobalamin bound vitamin B12 (holotranscobalamin); LDL, low-density lipoprotein; TfR, transferrin receptor; U–I, urinary iodine

**SUPPLEMENTARY TABLE 4** Sources of nutrients as daily mean proportions from each ingredient group based on 4-day food records for MEAT (*n* = 50) and LEGUME (*n* = 49) groups^a^ at the endpoint of the BeanMan 6-week RCT, where the diets between groups differed in proportions of red and processed meat (RPM) and legumes

| Ingredient group | MEAT | LEGUME |  | MEAT | LEGUME |  | MEAT | LEGUME |  | MEAT | LEGUME |
| --- | --- | --- | --- | --- | --- | --- | --- | --- | --- | --- | --- |
|  | Energy | |  | Protein | |  | Carbohydrates | |  | Fiber | |
|  | %, mean (SD) | |  | %, mean (SD) | |  | %, mean (SD) | |  | %, mean (SD) | |
| Vegetables | 3.3 (2.5) | 2.9 (2.2) |  | 2.8 (2.0) | 2.4 (1.7) |  | 4.6 (3.5) | 4.0 (3.5) |  | 16.9 (9.4) | 13.0 (7.9) |
| Nuts and seeds | 2.5 (3.9) | 3.1 (3.6) |  | 2.0 (3.1) | 2.8 (3.2) |  | 0.7 (1.9) | 0.8 (1.5) |  | 3.4 (6.5) | 3.3 (3.6) |
| Legumes | 0.1 (0.1) | 12.0 (4.4) |  | 0.1 (0.3) | 23.3 (9.4) |  | 0.1 (0.2) | 6.6 (4.8) |  | 0.1 (0.4) | 19.7 (10.5) |
| Pea and faba bean | 0.0 (0.0) | 11.4 (4.2) |  | 0.0 (0.0) | 22.7 (9.2) |  | 0.0 (0.0) | 6.2 (4.5) |  | 0.0 (0.2) | 18.7 (9.8) |
| Soy and other legumes | 0.1 (0.1) | 0.5 (1.2) |  | 0.1 (0.3) | 0.5 (1.1) |  | 0.1 (0.2) | 0.4 (0.9) |  | 0.1 (0.3) | 1.0 (2.6) |
| Potatoes | 4.2 (4.1) | 3.8 (3.2) |  | 2.4 (2.3) | 2.1 (1.8) |  | 7.4 (6.7) | 6.7 (5.2) |  | 4.5 (5.8) | 3.8 (3.7) |
| Fruits and berries | 5.2 (4.8) | 4.3 (4.2) |  | 1.4 (1.3) | 1.2 (1.2) |  | 9.5 (7.9) | 7.7 (6.8) |  | 14.7 (10.3) | 11.2 (10.2) |
| Cereals | 27.3 (7.8) | 26.2 (8.5) |  | 20.3 (6.3) | 20.8 (7.4) |  | 48.6 (13.0) | 46.4 (11.6) |  | 53.0 (13.9) | 43.0 (11.9) |
| Fat spreads, oils, and other fats | 12.0 (4.7) | 14.1 (5.2) |  | 0.2 (0.3) | 0.5 (1.1) |  | 0.2 (0.3) | 0.2 (0.2) |  | 0.1 (0.4) | 0.4 (1.2) |
| Fish and seafood | 3.1 (3.6) | 2.2 (2.4) |  | 7.7 (8.1) | 6.1 (6.1) |  | 0.4 (1.2) | 0.2 (0.7) |  | 0.6 (1.9) | 0.2 (1.0) |
| Red meat and processed red meat | 9.5 (4.2) | 1.3 (1.5) |  | 26.3 (10.9) | 4.6 (4.4) |  | 0.5 (1.1) | 0.2 (1.0) |  | 0.2 (0.5) | 0.0 (0.2) |
| Red meat | 5.3 (2.7) | 0.6 (1.5) |  | 18.4 (9.6) | 1.7 (3.8) |  | 0.2 (0.8) | 0.2 (1.0) |  | 0.1 (0.3) | 0.0 (0.2) |
| Processed red meat | 4.2 (2.9) | 0.7 (0.9) |  | 7.9 (4.4) | 2.9 (3.6) |  | 0.3 (0.7) | 0.0 (0.0) |  | 0.1 (0.4) | 0.0 (0.0) |
| White meat | 3.1 (4.2) | 2.4 (3.6) |  | 9.1 (11.2) | 7.0 (9.2) |  | 0.1 (0.2) | 0.1 (0.2) |  | 0.1 (0.4) | 0.0 (0.2) |
| Egg | 1.5 (1.8) | 1.2 (1.8) |  | 3.3 (3.8) | 2.5 (3.5) |  | 0.1 (0.1) | 0.0 (0.0) |  | 0.0 (0.0) | 0.0 (0.0) |
| Milk and dairy products | 14.6 (6.5) | 12.9 (6.8) |  | 19.7 (8.8) | 22.2 (11.3) |  | 7.5 (5.3) | 6.3 (4.9) |  | 0.7 (1.8) | 0.6 (1.6) |
| Plant-based dairy substitutes | 1.4 (2.1) | 2.0 (3.3) |  | 0.7 (1.2) | 0.8 (1.4) |  | 1.6 (3.0) | 2.1 (5.1) |  | 1.3 (2.2) | 1.7 (3.8) |
| Sugar, confectionery, and chocolate | 6.2 (5.3) | 5.7 (5.3) |  | 1.3 (1.4) | 1.1 (1.2) |  | 10.6 (10.0) | 9.7 (9.4) |  | 2.1 (2.1) | 1.3 (1.4) |
| Other beverages and products | 5.9 (4.0) | 6.0 (5.2) |  | 2.6 (1.6) | 2.5 (1.8) |  | 8.1 (5.3) | 8.9 (7.3) |  | 2.3 (2.1) | 2.0 (1.9) |

^a^The MEAT group followed a diet including 760 g/wk (cooked weight) of red and processed meat (beef and pork), accounting for 25% of the total protein intake. The LEGUME group followed a diet including legume-based products containing protein equivalent to 560 g/wk (cooked weight) of red meat, plus 200 g/wk of red and processed meat (beef and pork); legumes accounted for 20% and red and processed meat 5% of the total protein intake

**SUPPLEMENTARY TABLE 5** Daily energy-yielding nutrient intakes based on 4-day food records and daily protein urinary excretion for MEAT and LEGUME groups^a^ in the BeanMan 6-week RCT

|  | MEAT (*n* = 51) | LEGUME (*n* = 50) |  | | MEAT (*n* = 50) | | LEGUME (*n* = 49) | *P* value between groups, endpoint^b^ | |  |
| --- | --- | --- | --- | --- | --- | --- | --- | --- | --- | --- |
|  | Baseline (week 0)  Mean (SD) | |  |  | Endpoint (week 6)  Mean (SD) | | |  |  |  |
|  |  |  |  |  |  |  |  |  |  |  |
| Energy intake, MJ | 10.8 (2.3) | 11.9 (2.4) | |  | | 10.4 (1.8) | 11.4 (2.2) | | **0.022** | |
| Protein, E% | 16.6 (2.6) | 17.0 (2.9) | |  | | 17.5 (2.8)^c^ | 16.9 (2.9) | | 0.230 | |
| Protein, g | 105.4 (28.3) | 119.7 (35.0) | |  | | 107.2 (24.4) | 112.0 (22.7) | | 0.267 | |
| Protein, urinary excretion^d^, g | 101.7 (26.4) | 102.7 (26.6) | |  | | 111.2 (35.7) | 104.1 (25.9) | | 0.234 | |
| Plant-source protein, %/animal-source protein^e^, % | 40/60 (14.0) | 42/58 (14.4) | |  | | 34/66 (8.8) | 58/42 (13.5) | | – | |
| Carbohydrates, E% | 41.3 (5.8) | 41.0 (5.4) | |  | | 41.0 (6.3) | 40.5 (6.6) | | 0.655 | |
| Carbohydrates, g | 258.8 (63.2) | 283.0 (64.8) | |  | | 247.8 (57.8) | 270.0 (70.0) | | 0.123 | |
| Fiber, E% | 2.1 (0.7) | 2.1 (0.7) | |  | | 2.0 (0.6) | 2.4 (0.6)^c^ | | **0.006** | |
| Fiber, g | 28.5 (11.5) | 31.7 (11.5) | |  | | 26.6 (9.0) | 33.9 (11.7)^c^ | | **<0.001** | |
| Fiber, g/MJ | 2.7 (0.9) | 2.7 (0.9) | |  | | 2.6 (0.7) | 2.9 (0.7)^c^ | | **0.006** | |
| Fat, total, E% | 37.9 (5.4) | 38.0 (5.8) | |  | | 37.7 (5.9) | 38.6 (6.2) | | 0.494 | |
| Fat, total, g | 110.9 (29.1) | 123.1 (34.6) | |  | | 106.0 (23.8) | 120.0 (33.8) | | **0.034** | |
| Monounsaturated fatty acids, E% | 14.1 (2.9) | 14.4 (2.4) | |  | | 14.3 (3.1) | 15.2 (2.9) | | 0.092 | |
| Polyunsaturated fatty acids, total, E% | 7.3 (2.3) | 7.4 (2.0) | |  | | 7.0 (1.8) | 8.4 (2.0)^c^ | | **<0.001** | |
| n-3 PUFAs, E% | 1.7 (0.6) | 1.7 (0.6) | |  | | 1.6 (0.7) | 2.0 (0.5)^c^ | | **<0.001** | |
| α-linolenic acid, E% | 1.3 (0.5) | 1.3 (0.4) | |  | | 1.2 (0.5) | 1.5 (0.5)^c^ | | **<0.001** | |
| n-6 PUFAs, E% | 5.4 (1.9) | 5.5 (1.6) | |  | | 5.2 (1.4) | 6.3 (1.7)^c^ | | **<0.001** | |
| linoleic acid, E% | 5.1 (1.9) | 5.1 (1.6) | |  | | 4.9 (1.3) | 5.6 (1.8) | | **0.041** | |
| Saturated fatty acids, E% | 12.7 (3.0) | 12.5 (3.5) | |  | | 12.7 (3.1) | 11.1 (3.0)^c^ | | **0.012** | |
| Trans fatty acids, g | 1.0 (0.6) | 1.1 (0.5) | |  | | 1.0 (0.4) | 0.9 (0.5)^c^ | | 0.191 | |
| Cholesterol, mg | 325 (146) | 322 (143) | |  | | 338 (136) | 256 (179)^c^ | | **<0.001**^4^ | |
| Alcohol^f^, E% | 2.1 (2.8) | 1.9 (2.4) | |  | | 1.8 (2.6) | 1.7 (2.6) | | 0.910^g^ | |

^a^The MEAT group followed a diet including 760 g/wk (cooked weight) of red and processed meat (beef and pork), accounting for 25% of the total protein intake. The LEGUME group followed a diet including legume-based products containing protein equivalent to 560 g/wk (cooked weight) of red meat, plus 200 g/wk of red and processed meat (beef and pork); legumes accounted for 20% and red and processed meat 5% of the total protein intake

^b^Two-sided *P* from independent samples *t*-test except for protein, urinary excretion *P* from ANCOVA adjusted for baseline with Bonferroni correction. All variables except alcohol were log10-transformed for testing. Bold means that the *P* is <0.05

^c^Statistically significant (two-sided *P* <0.05) difference within a group between log10-transformed baseline and endpoint values according to paired samples *t*-test

^d^Estimated using the formula by Maroni et al. (1985). MEAT group endpoint *n* = 49, LEGUME group baseline/endpoint *n* = 51/50; ANCOVA analysis MEAT group *n* = 49, LEGUME group *n* = 50

^e^Calculated from food weight. Ingredient groups for plant-source protein: vegetables, nuts and seeds, pea and faba bean, soy and other legumes, potatoes, fruits and berries, cereals, plant-based dairy substitutes, sugar, confectionery, and chocolate, other beverages; for animal-source protein: fish and seafood, red meat, processed red meat, white meat, egg, milk and dairy products

^f^Mean values for all participants including non-consumers baseline/endpoint MEAT group *n* = 18/23, LEGUME group *n* = 19/21

^g^*P* from Mann-Whitney U test

E%, percentage of energy; PUFA, polyunsaturated fatty acids

**SUPPLEMENTARY TABLE 6** Daily micronutrient intakes for MEAT and LEGUME groups^a^ in the BeanMan 6-week RCT

|  | MEAT (*n* = 51) | LEGUME (*n* = 50) |  | MEAT (*n* = 50) | | LEGUME (*n* = 49) | *P* value between groups, endpoint^b^ | |  |
| --- | --- | --- | --- | --- | --- | --- | --- | --- | --- |
|  | Baseline (week 0)  Mean (SD) | |  | Endpoint (week 6)  Mean (SD) | | |  |  |  |
|  |  |  |  |  |  |  |  |  |  |
| Vitamin B12, µg | 7.41 (7.60) | 6.58 (3.00) |  | 7.14 (3.83) | 4.72 (2.50)^c^ | | | **<0.001** | |
| Vitamin B12, µg/MJ | 0.69 (0.77) | 0.56 (0.25) |  | 0.67 (0.30) | 0.41 (0.22)^c^ | | | **<0.001** | |
| Iodine, µg | 242 (82.3) | 273 (97.8) |  | 245 (71.2) | 246 (76.3)^c^ | | | 0.887 | |
| Iodine, µg/MJ | 22.5 (5.57) | 22.8 (6.61) |  | 23.5 (5.03) | 21.6 (5.66) | | | 0.057 | |
| Iron, mg | 15.1 (4.98) | 17.6 (6.81) |  | 13.8 (3.00) | 21.3 (5.01)^c^ | | | **<0.001** | |
| Iron, mg/MJ | 1.40 (0.35) | 1.47 (0.44) |  | 1.33 (0.21) | 1.89 (0.41)^c^ | | | **<0.001** | |
| Plant-source iron^d^, mg | 11.5 (4.78) | 14.3 (6.91) |  | 9.8 (2.94)^c^ | 19.7 (4.93)^c^ | | | **<0.001** | |
| Animal-source iron^d^, mg | 3.77 (2.76) | 3.70 (2.27) |  | 3.94 (1.36)^c^ | 1.59 (1.01)^c^ | | | **<0.001** | |
| Ratio of plant-source to animal-source iron^d^, mg/mg | 6.33 (14.4) | 9.82 (20.5) |  | 3.00 (2.29)^c^ | 22.6 (34.6)^c^ | | | **<0.001** | |
| Vitamin C, mg | 138 (90.1) | 144 (74.6) |  | 135 (89.1) | 155 (94.3) | | | 0.233 | |
| Vitamin C, mg/MJ | 12.7 (7.09) | 12.2 (6.41) |  | 13.0 (8.06) | 13.3 (7.53) | | | 0.521 | |
| Molar ratio of vitamin C to iron | 2.9:1 | 2.7:1 |  | 3.0:1 | 2.3:1^c^ | | | 0.065 | |

^a^The MEAT group followed a diet including 760 g/wk (cooked weight) of red and processed meat (beef and pork), accounting for 25% of the total protein intake. The LEGUME group followed a diet including legume-based products containing protein equivalent to 560 g/wk (cooked weight) of red meat, plus 200 g/wk of red and processed meat (beef and pork); legumes accounted for 20% and red and processed meat 5% of the total protein intake

^b^Two-sided *P* from independent samples *t*-test. All variables were log10-transformed for testing. Bold means that the *P* is <0.05.

^c^Statistically significant (two-sided *P* <0.05) difference within a group between log10-transformed baseline and endpoint values, according to paired samples *t*-test

^d^Ingredient groups for plant-source iron: vegetables, nuts and seeds, pea and faba bean, soy and other legumes, potatoes, fruits and berries, cereals, plant-based dairy substitutes, sugar, confectionery, and chocolate, other beverages; for animal-source protein: fish and seafood, red meat, processed red meat, white meat, egg, milk and dairy products. The amount of plant-source and animal-source iron at the ingredient level before cooking

**SUPPLEMENTARY TABLE 7** Sources of nutrients as daily mean proportions from each ingredient group based on 4-day food records for MEAT (*n* = 50) and LEGUME (*n* = 49) groups^a^ at the endpoint of the BeanMan 6-week RCT, where the diets between groups differed in proportions of red and processed meat (RPM) and legumes

| Ingredient group | MEAT | LEGUME |  | MEAT | LEGUME |  | MEAT | LEGUME |  | MEAT | LEGUME |
| --- | --- | --- | --- | --- | --- | --- | --- | --- | --- | --- | --- |
|  | Fat, total | |  | Saturated fatty acids | |  | PUFAs | |  | n-3 PUFAs | |
|  | %, mean (SD) | |  | %, mean (SD) | |  | %, mean (SD) | |  | %, mean (SD) | |
| Vegetables | 1.6 (2.9) | 1.4 (1.6) |  | 0.7 (1.2) | 0.7 (0.9) |  | 3.5 (8.4) | 2.2 (3.1) |  | 4.0 (9.1) | 2.1 (2.7) |
| Nuts and seeds | 4.8 (7.1) | 5.5 (6.2) |  | 2.2 (3.8) | 2.8 (3.6) |  | 8.3 (11.9) | 8.4 (10.3) |  | 3.9 (9.5) | 4.4 (8.4) |
| Legumes | 0.0 (0.0) | 13.6 (4.7) |  | 0.0 (0.0) | 4.8 (3.2) |  | 0.0 (0.0) | 18.1 (7.1) |  | 0.0 (0.0) | 23.6 (9.9) |
| Pea and faba bean | 0.0 (0.0) | 13.0 (4.7) |  | 0.0 (0.0) | 4.7 (3.2) |  | 0.0 (0.0) | 17.1 (7.1) |  | 0.0 (0.0) | 22.3 (10.0) |
| Soy and other legumes | 0.0 (0.0) | 0.6 (1.7) |  | 0.0 (0.0) | 0.2 (0.4) |  | 0.0 (0.0) | 1.0 (2.7) |  | 0.0 (0.0) | 1.3 (3.6) |
| Potatoes | 2.0 (4.0) | 1.6 (3.0) |  | 1.8 (3.8) | 1.5 (3.7) |  | 2.2 (4.7) | 1.9 (3.2) |  | 2.2 (4.1) | 1.9 (3.0) |
| Fruits and berries | 1.7 (3.1) | 1.8 (4.3) |  | 1.0 (2.5) | 1.0 (3.1) |  | 1.2 (1.4) | 0.9 (1.9) |  | 1.8 (1.8) | 1.1 (1.6) |
| Cereals | 8.7 (4.8) | 8.4 (5.7) |  | 5.5 (4.1) | 6.8 (6.7) |  | 14.9 (7.3) | 12.3 (6.5) |  | 9.2 (7.3) | 6.7 (5.3) |
| Fat spreads, oils, and other fats | 31.3 (11.0) | 35.6 (10.4) |  | 20.6 (9.8) | 28.4 (12.7) |  | 42.0 (13.9) | 40.9 (12.6) |  | 46.3 (18.8) | 40.8 (15.5) |
| Fish and seafood | 4.2 (5.4) | 3.0 (3.8) |  | 2.5 (3.3) | 1.9 (2.6) |  | 6.8 (8.5) | 4.4 (5.3) |  | 16.9 (18.4) | 11.3 (13.7) |
| Red meat and processed red meat | 12.1 (5.9) | 1.2 (1.7) |  | 14.2 (7.4) | 1.4 (1.8) |  | 7.4 (4.7) | 0.8 (1.6) |  | 4.1 (3.2) | 0.6 (1.8) |
| Red meat | 5.2 (2.9) | 0.7 (1.6) |  | 6.2 (4.0) | 0.7 (1.5) |  | 2.6 (2.2) | 0.6 (1.7) |  | 1.9 (2.5) | 0.4 (1.8) |
| Processed red meat | 6.9 (5.0) | 0.5 (0.9) |  | 8.0 (5.8) | 0.7 (1.2) |  | 4.7 (3.8) | 0.3 (0.5) |  | 2.2 (1.9) | 0.1 (0.2) |
| White meat | 3.4 (4.9) | 2.8 (5.0) |  | 2.6 (4.4) | 2.6 (4.7) |  | 4.1 (5.7) | 2.9 (5.4) |  | 3.4 (5.6) | 1.8 (5.1) |
| Egg | 2.5 (2.7) | 2.0 (3.0) |  | 2.1 (2.8) | 1.9 (3.2) |  | 2.0 (2.2) | 1.3 (1.9) |  | 1.3 (1.8) | 0.7 (1.1) |
| Milk and dairy products | 21.0 (10.1) | 16.7 (9.1) |  | 38.0 (14.5) | 36.1 (16.1) |  | 3.6 (3.0) | 2.3 (1.7) |  | 3.3 (3.2) | 1.9 (1.6) |
| Plant-based dairy substitutes | 1.7 (2.4) | 2.5 (4.1) |  | 1.7 (3.6) | 3.1 (5.9) |  | 1.6 (2.6) | 2.1 (3.5) |  | 1.3 (2.9) | 1.9 (3.6) |
| Sugar, confectionery, and chocolate | 4.3 (4.2) | 3.4 (4.1) |  | 6.4 (6.4) | 6.5 (8.5) |  | 1.9 (2.3) | 1.0 (1.6) |  | 1.6 (2.4) | 0.7 (1.6) |
| Other beverages and products | 0.7 (0.7) | 0.5 (0.5) |  | 0.6 (1.0) | 0.4 (0.5) |  | 0.6 (0.7) | 0.4 (0.6) |  | 0.8 (0.9) | 0.5 (0.8) |

^a^The MEAT group followed a diet including 760 g/wk (cooked weight) of red and processed meat (beef and pork), accounting for 25% of the total protein intake. The LEGUME group followed a diet including legume-based products containing protein equivalent to 560 g/wk (cooked weight) of red meat, plus 200 g/wk of red and processed meat (beef and pork); legumes accounted for 20% and red and processed meat 5% of the total protein intake

**SUPPLEMENTARY TABLE 8** Sources of nutrients as daily mean proportions from each ingredient group based on 4-day food records for MEAT (*n* = 50) and LEGUME (*n* = 49) groups^a^ at the endpoint of the BeanMan 6-week RCT, where the diets between groups differed in proportions of red and processed meat (RPM) and legumes

| Ingredient group | MEAT | LEGUME |  | MEAT | LEGUME |  |
| --- | --- | --- | --- | --- | --- | --- |
|  | n-6 PUFAs | |  | Cholesterol | |  |
|  | %, mean (SD) | |  | %, mean (SD) | |  |
| Vegetables | 3.5 (8.7) | 2.2 (3.4) |  | 1.1 (3.9) | 1.1 (2.4) |  |
| Nuts and seeds | 9.4 (12.6) | 9.6 (11.3) |  | 0.0 (0.0) | 0.0 (0.0) |  |
| Legumes | 0.0 (0.0) | 16.7 (6.6) |  | 0.0 (0.0) | 0.4 (0.4) |  |
| Pea and faba bean | 0.0 (0.0) | 15.8 (6.5) |  | 0.0 (0.0) | 0.3 (0.3) |  |
| Soy and other legumes | 0.0 (0.0) | 0.9 (2.5) |  | 0.0 (0.0) | 0.1 (0.2) |  |
| Potatoes | 2.1 (5.1) | 2.0 (3.6) |  | 0.5 (1.3) | 0.6 (2.2) |  |
| Fruits and berries | 1.1 (1.5) | 0.9 (2.0) |  | 0.0 (0.1) | 0.1 (0.1) |  |
| Cereals | 17.3 (8.3) | 14.2 (7.5) |  | 2.9 (3.9) | 3.2 (5.1) |  |
| Fat spreads, oils, and other fats | 41.8 (14.4) | 41.3 (13.4) |  | 7.2 (5.6) | 10.8 (9.4) |  |
| Fish and seafood | 2.9 (4.4) | 1.8 (2.4) |  | 9.4 (10.1) | 10.4 (10.9) |  |
| Red meat and processed red meat | 7.6 (4.9) | 0.8 (1.6) |  | 25.6 (13.3) | 8.8 (14.6) |  |
| Red meat | 2.2 (2.0) | 0.5 (1.6) |  | 16.4 (10.3) | 2.0 (5.4) |  |
| Processed red meat | 5.4 (4.4) | 0.3 (0.5) |  | 9.2 (6.0) | 6.7 (14.5) |  |
| White meat | 4.4 (6.1) | 3.3 (5.8) |  | 9.8 (11.8) | 10.9 (16.8) |  |
| Egg | 2.3 (2.5) | 1.5 (2.3) |  | 23.6 (20.5) | 24.0 (23.1) |  |
| Milk and dairy products | 3.1 (2.6) | 1.9 (1.4) |  | 18.7 (10.0) | 28.8 (18.9) |  |
| Plant-based dairy substitutes | 1.8 (2.9) | 2.2 (3.6) |  | 0.0 (0.0) | 0.1 (0.2) |  |
| Sugar, confectionery, and chocolate | 2.0 (2.4) | 1.1 (1.7) |  | 1.0 (2.1) | 0.7 (1.4) |  |
| Other beverages and products | 0.6 (0.7) | 0.4 (0.6) |  | 0.2 (0.4) | 0.2 (0.4) |  |

^a^The MEAT group followed a diet containing 760 g/wk (cooked weight) of RPM, accounting for 25% of the total protein intake. The LEGUME group followed a diet including legume-based products containing protein equivalent to 560 g/wk (cooked weight) of red meat, plus 200 g/wk of red and processed meat (beef and pork); legumes accounted for 20% and red and processed meat 5% of the total protein intake

**SUPPLEMENTARY TABLE 9** Sources of nutrients as daily mean proportions from each ingredient group based on 4-day food records for MEAT (*n* = 50) and LEGUME (*n* = 49) groups^a^ at the endpoint of the BeanMan 6-week RCT, where the diets between groups differed in proportions of red and processed meat (RPM) and legumes

| Ingredient group | MEAT | LEGUME |  | MEAT | LEGUME |  | MEAT | LEGUME |  |
| --- | --- | --- | --- | --- | --- | --- | --- | --- | --- |
|  | Vitamin B12 | |  | Iodine^b^ | |  | Iron | |  |
|  | %, mean (SD) | |  | %, mean (SD) | |  | %, mean (SD) | |  |
| Vegetables | 0.2 (0.8) | 0.1 (0.5) |  | 2.1 (2.6) | 1.9 (2.3) |  | 9.3 (6.1) | 5.1 (3.1) |  |
| Nuts and seeds | 0.0 (0.0) | 0.0 (0.0) |  | 0.3 (0.6) | 0.5 (0.9) |  | 3.4 (5.1) | 3.4 (3.9) |  |
| Legumes | 0.0 (0.0) | 0.0 (0.0) |  | 0.0 (0.0) | 9.4 (6.6) |  | 0.7 (1.6) | 41.5 (14.4) |  |
| Pea and faba bean | 0.0 (0.0) | 0.0 (0.0) |  | 0.0 (0.0) | 8.5 (6.1) |  | 0.0 (0.1) | 40.3 (14.1) |  |
| Soy and other legumes | 0.0 (0.0) | 0.0 (0.0) |  | 0.0 (0.0) | 0.9 (2.5) |  | 0.6 (1.5) | 1.2 (2.0) |  |
| Potatoes | 0.4 (1.7) | 0.8 (4.3) |  | 1.4 (1.9) | 1.4 (2.0) |  | 4.7 (4.5) | 3.3 (2.6) |  |
| Fruits and berries | 0.0 (0.0) | 0.0 (0.0) |  | 0.8 (0.7) | 0.7 (0.7) |  | 6.8 (5.6) | 4.1 (4.1) |  |
| Cereals | 0.9 (1.6) | 1.4 (2.7) |  | 16.6 (7.9) | 16.4 (6.9) |  | 34.1 (11.5) | 26.3 (11.6) |  |
| Fat spreads, oils, and other fats | 0.7 (1.5) | 1.3 (2.0) |  | 1.5 (1.5) | 1.6 (1.6) |  | 0.5 (0.7) | 0.6 (1.2) |  |
| Fish and seafood | 22.4 (22.0) | 24.7 (23.4) |  | 9.6 (12.5) | 7.7 (9.4) |  | 2.9 (3.9) | 1.3 (1.9) |  |
| Red meat and processed red meat | 30.9 (18.9) | 5.9 (9.9) |  | 2.8 (1.8) | 0.4 (0.4) |  | 17.6 (9.6) | 1.2 (1.2) |  |
| Red meat | 16.7 (10.8) | 1.4 (3.4) |  | 1.3 (0.8) | 0.2 (0.4) |  | 10.2 (6.7) | 0.3 (0.8) |  |
| Processed red meat | 14.2 (14.5) | 4.5 (9.9) |  | 1.5 (1.7) | 0.2 (0.3) |  | 7.5 (7.2) | 0.9 (1.1) |  |
| White meat | 5.8 (7.8) | 6.2 (10.0) |  | 2.8 (4.7) | 2.3 (3.9) |  | 2.5 (3.6) | 1.5 (2.4) |  |
| Egg | 9.5 (11.4) | 10.2 (12.1) |  | 4.9 (5.6) | 3.8 (5.0) |  | 3.4 (3.8) | 2.0 (2.8) |  |
| Milk and dairy products | 24.0 (13.6) | 41.7 (23.1) |  | 20.8 (11.1) | 22.6 (13.8) |  | 2.4 (1.4) | 1.6 (1.2) |  |
| Plant-based dairy substitutes | 2.7 (5.3) | 6.3 (12.8) |  | 3.3 (7.2) | 2.4 (6.6) |  | 2.1 (3.3) | 1.8 (3.8) |  |
| Sugar, confectionery, and chocolate | 0.3 (0.7) | 0.2 (0.4) |  | 2.3 (2.5) | 2.3 (3.1) |  | 3.2 (3.2) | 2.3 (2.9) |  |
| Other beverages and products | 2.1 (10.2) | 1.1 (4.7) |  | 30.9 (11.5)^c^ | 26.6 (15.9)^c^ |  | 6.5 (4.2) | 4.1 (3.1) |  |

^a^The MEAT group followed a diet including 760 g/wk (cooked weight) of red and processed meat (beef and pork), accounting for 25% of the total protein intake. The LEGUME group followed a diet including legume-based products containing protein equivalent to 560 g/wk (cooked weight) of red meat, plus 200 g/wk of red and processed meat (beef and pork); legumes accounted for 20% and red and processed meat 5% of the total protein intake

^b^Iodized salt that was disaggregated from composite dishes was included in the ingredient group “other beverages and products”. However, iodized salt was not disaggregated from all industrial bakery products and foods fortified with iodized salt, e.g., “cereals” ingredient group comprises cereals as such, which do not contain much iodine, but also foods made from cereals, which contain iodized salt added during industrial processing

^c^Includes iodized salt

**SUPPLEMENTARY FIGURE 1** Daily intake of vitamin B12 of healthy men at baseline and endpoint of the BeanMan 6-week RCT, where the diets of intervention groups differed in proportions of red and processed meat (RPM) and legumes. (A) The MEAT group (*n* = baseline 51/endpoint 50) followed a diet including 760 g/wk (cooked weight) of red and processed meat (beef and pork), accounting for 25% of the total protein intake. (B) The LEGUME group (*n* = baseline 50/endpoint 49) followed a diet including legume-based products containing protein equivalent to 560 g/wk (cooked weight) of red meat, plus 200 g/wk of red and processed meat (beef and pork); legumes accounted for 20% and red and processed meat 5% of the total protein intake. Boxplots represent 25^th^ percentile, median (line), mean (square), 75^th^ percentile, and whiskers (range within 1.5 IQR) with individual values connected between baseline and endpoint. The line at 4 µg/d presents adequate intake (AI) (Blomhoff et al. 2023)

**SUPPLEMENTARY FIGURE 2** Transcobalamin bound vitamin B12 (holoTC) concentrations of healthy men at baseline and endpoint of the BeanMan 6-week RCT, where the diets of intervention groups differed in proportions of red and processed meat (RPM) and legumes. (A) The MEAT group (*n* = 51) followed a diet including 760 g/wk (cooked weight) of red and processed meat (beef and pork), accounting for 25% of the total protein intake. (B) The LEGUME group (*n* = baseline 50/endpoint 51) followed a diet including legume-based products containing protein equivalent to 560 g/wk (cooked weight) of red meat, plus 200 g/wk of red and processed meat (beef and pork); legumes accounted for 20% and red and processed meat 5% of the total protein intake. Boxplots represent 25^th^ percentile, median (line), mean (square), 75^th^ percentile, and whiskers (range within 1.5 IQR) with individual values connected between baseline and endpoint. The line at 35 pmol/L presents deficiency and at 50 pmol/L marginal status (Allen et al. 2018)
